# Complete degradation of polycyclic antibiotic methacycline by a micro/nanostructured biogenic Mn oxide composite from engineered Mn(II)-oxidizing *Pseudomonas* sp. MB04B

**DOI:** 10.1128/spectrum.01611-24

**Published:** 2025-05-16

**Authors:** Jie Zeng, Zhenghu Tong, Zhi Li, Yongxuan Liu, Li Xie, Tan Wang, Shiwei Li, Lin Li

**Affiliations:** 1 National Key Laboratory of Agricultural Microbiology, College of Life Science and Technology, Huazhong Agricultural University, Wuhan, China; University of Mississippi, University, Mississippi, USA

**Keywords:** biogenic Mn oxide, methacycline degradation, gene knockout, *Pseudomonas*, bioremediation

## Abstract

The misuse and improper disposal of methacycline (MTC), a widely used broad-spectrum antibiotic in human clinical settings and livestock production, poses significant threats to both human health and the ecological environment. In this study, a wild-type Mn(II)-oxidizing *Pseudomonas* strain MB04B was modified through multiple gene deletions, leading to a maximum 35% increase in Mn oxide deposit amount (MnODA) in the engineered strain MB04R-14, and accelerated formation of biogenic Mn oxide (BMO) aggregates, which exhibited the capability to degrade and detoxify MTC completely. After constructing a mini-Tn5 transposon insertion mutant library and screening for MnODA-increased mutants, a total of 10 target genes located in the corresponding mutant loci were identified using the high-efficiency thermal asymmetric interlaced PCR (hiTAIL-PCR) method. These genes were systematically knocked out singly or in multiple combinations, and the highest MnODA-promoted mutant (MB04R-14) was obtained, in which seven genes were knocked out. Following the characterization of the BMO aggregate complex formed in MB04R-14 as a micro-/nanostructured ramsdellite (MnO_2_) composite through means of several analysis methods, the complex was assessed for MTC degradation under laboratory trials. Complete MTC degradation was revealed after 24 h of treatment with the BMO complex, and the metal ions Mg^2+^, Cu^2+^, Ni^2+^, and Co^2+^ significantly inhibited MTC degradation efficiency. Liquid chromatography-mass spectrometry identified three intermediates in the degradation pathway, and a possible degradation-metabolic pathway of MTC by the BMO complex was proposed. Finally, the residual antibiotic activity, continuous degradation cycle performance, and treatment of MTC-containing hospital wastewater were evaluated.

**IMPORTANCE:**

Due to the common usage and recalcitrance to degradation, methacycline is often found in various surface water and wastewater as a persistent antibiotic toxicant, posing significant risks to the environment and public health. By engineering a *Pseudomonas* strain, we developed a dynamic oxidative composite comprising engineered *Pseudomonas* cells and biogenic Mn oxides. This system not only enhances oxidative capacities but also accelerates the formation of biogenic Mn oxides, leading to the complete degradation of methacycline. The findings highlight the potential of engineered *Pseudomonas* strain as a sustainable solution for mitigating antibiotic pollution, thereby contributing to cleaner water resources and protecting ecosystems.

## INTRODUCTION

Antibiotics play a crucial role in both human clinical and livestock production. They are also frequently incorporated in feed to bolster the resistance of livestock and poultry against pathogenic microorganisms (1, 2). In China, the vast population and extensive livestock and poultry farming contribute to the country’s high production and consumption of antibiotics. A variety of antibiotics, including β-lactams, macrocyclic lactones, sulfonamides, tetracyclines, and fluoroquinolones, are among the most widely used globally, with more than half of these antibiotics being used in animal husbandry (3, 4). However, both human and animal bodies are unable to fully absorb and metabolize ingested antibiotics. It is estimated that 30% to 90% of these antibiotics are excreted in urine and feces, eventually entering water and soil environments. This leads to the gradual accumulation of antibiotics in these ecosystems (5). Consequently, elevated levels of antibiotics and drug residues have been consistently identified in various water bodies across China over the decades. For an example, in a study of 18 water source samples collected from the Wuhan section of the Yangtze River, a total of 14 antibiotics were detected. The highest concentration recorded was found for tetracyclines at 1,708.33 ng/L (6, 7). These residual antibiotics in the environment retain their activity (8), potentially affecting various soil and aquatic organisms by altering evolutionary pathways (9), disrupting community distribution (10), diminishing biodiversity (11, 12), and increasing antibiotic-resistant infection through the enrichment of resistant pathogens (13). Additionally, antibiotics that leach into soil and water can be taken up by crops cultivated in these environments. These antibiotics can accumulate in the food chain and eventually enter the human body, posing significant health risks, including the potential development of antibiotic-resistant infections in humans (9, 13).

Methacycline (MTC) is a broad-spectrum antibiotic belonging to the tetracycline class, characterized by a hydrogenated tetraphenyl-ring perhydrophenanthrene structure. It is typically synthesized through semi-synthetic processes from oxytetracycline, which shares similar antibacterial properties with tetracycline. MTC effectively inhibits a broad spectrum of gram-positive and gram-negative bacteria, including *Rickettsia*, *Mycoplasma*, and *Chlamydia*, with activity even against strains resistant to tetracycline (14, 15). In human clinical practice, MTC hydrochloride tablets are extensively used to treat diseases such as typhus, acne, salpingitis, and infections caused by *Mycoplasma* and *Chlamydia*. Additionally, they are commonly used as veterinary medicines and growth-promoting agents in animal husbandry and pet care, as exemplified by practices in countries including the USA where MTCs are added to animal feed at subtherapeutic levels—doses lower than those required for treating bacterial infections—in order to enhance the overall health and growth performance of livestock (16, 17). Moreover, the chemical stability of MTC surpasses that of other tetracycline-class antibiotics, allowing it to maintain a prolonged circulation duration in the bloodstream and persist in the environment for an extended period. This stability makes MTC particularly effective in treating infections over longer periods. However, this has also led to the accumulation of residual MTC, not only in animal waste but also in animal tissues. Previous investigations have recorded MTC concentrations of 159.9 µg/kg in honey (18), and the highest concentration of MTC in livestock and poultry excrement reached up to 14.28 mg/kg (19). The presence of such residues in the environment is a growing concern, especially considering the potential risks of contamination in food and water sources. Unfortunately, the complex polycyclic structure of MTC makes traditional physiochemical methods for removing antibiotic residues, such as adsorption, coagulation, membrane filtration, and chemical oxidation, generally considered costly and ineffective (20–24). Therefore, there is an imperative demand for innovative and sustainable approaches to address the challenges posed by MTC antibiotic residues in the environment.

Recently, the potential applicability of biogenic Mn oxides (BMOs) in the remediation of organic and metal pollutants has drawn increasing attention due to their promising oxidative and adsorptive properties (25–27). BMOs possess a high redox potential and function as a naturally occurring potent oxidizing agent (28). BMOs are formed during Mn mineralization, where Mn(II) ions are swiftly oxidized into Mn(III) and Mn(IV) oxides, primarily by Mn(II)-oxidizing microorganisms in the natural environment (29). BMOs are predominantly composed of Mn(IV) with a minor content of Mn(III), forming hexagonally symmetrical weak crystal layers closest to chemically synthesized δ-MnO_2_ and birnessite (30, 31), with a relatively high surface area, unordered accumulation along the C axes, high valences, and several vacancies in their octahedral structures (32). These unique features allow BMOs to effectively facilitate the degradation of polycyclic aromatic organic substances, such as antibiotics, through various metabolic pathways (25–27), thereby facilitating the degradation of polycyclic aromatic organic substances through various metabolic pathways. For instance, Mn oxides stimulate the production of intracellular reactive oxygen species, including hydroxyl radicals (⋅OH) and superoxide radicals (O_2_⋅^−^), which break benzene rings and degrade certain aromatic organic pollutants (33). Several previous investigations have demonstrated the capability of Mn oxides to completely degrade and detoxify certain antibiotics, such as tetracyclines, fluoroquinolones, sulfonamides, and cephalosporins, among others (34–39). These studies highlight the promising potential of Mn oxides in mitigating the environmental impact of antibiotic residues by breaking down complex antibiotic molecules into less harmful substances. However, to date, there has been no report on the degradation of MTC by BMOs specifically.

In previous investigations, we have demonstrated that certain Mn(II)-oxidizing bacteria are capable of degrading aromatic pollutants, such as bisphenol A and nonylphenol (40) and diclofenac (41) by forming BMO composites. However, the Mn(II)-oxidizing activity of these bacterial strains has remained relatively low, often requiring an extended period of continuous cultivation to form the BMO complexes. In this study, through genetic modification of a wild-type *Pseudomonas* strain (namely MB04B), we successfully engineered a more efficient Mn(II)-oxidizing system capable of rapidly and completely degrading MTC. By identifying key genes involved in Mn oxide formation, characterizing the resulting BMO complexes, and validating the degradation and detoxification activities of BMOs toward MTC, this study aims to develop a sustainable, biologically driven remediation approach for polycyclic aromatic antibiotic pollutants.

## RESULTS

### Construction of the engineered strain MB04R-14 for accelerated formation of Mn oxides and enhanced MnODA production

**Fig 1:**
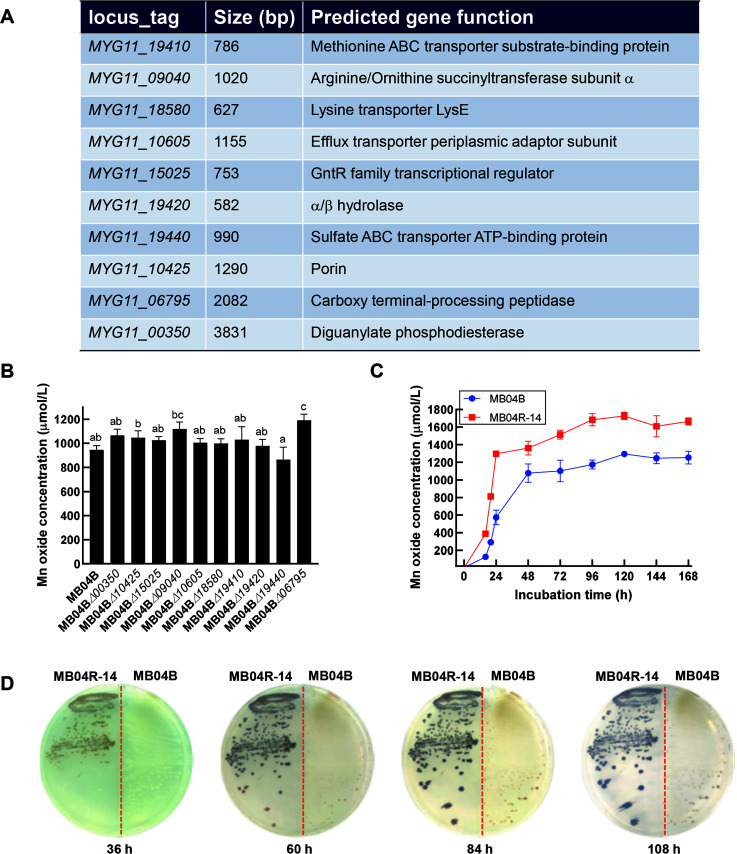
The predicted function of 10 genes in the loci of mini-Tn5 mutagenesis involved in Mn(II)-oxidizing activity promotion in *Pseudomonas* sp. MB04B (**A**); measurement of cell-suspension MnODA in different gene‐knockout mutant strains (**B**); time course of cell-suspension MnODA of the engineered strain MB04R-14 (**C**); and photographs of MB04R-14 and MB04B colonies cultured on the Lept plates (**D**). Means followed by different letters in a column are significantly different (*P* < 0.05) according to the Student–Newman–Keuls test.

To investigate the genes affecting Mn(II)-oxidizing activity in the genome of a wild-type *Pseudomonas* sp. MB04B isolated from a soilborne Fe–Mn nodule (42) with relatively high Mn(II)-oxidizing activity (abbreviated as MnODA), a mini-scale transposon mutation library comprising 3,562 mutants was constructed using the mini-Tn5 transposon random mutagenesis strategy (Fig. S1). Subsequently, the MnODA of these mutant strains was determined using the standard LBB (leucoberbelin blue) method. Ten mutant strains exhibiting significantly increased MnODA were identified (Fig. S2). The mini-Tn5 insertion sites in these 10 mutant strains were further probed using the high-efficiency thermal asymmetric interlaced PCR (hiTAIL-PCR) method. Following the second and third rounds of hiTAIL-PCR amplifications, the amplified products were electrophoretically separated. Bands derived from the third-round (Fig. S3, indicated by the red arrow) amplifications, smaller by approximately 100 to 200 bp compared to those of the second rounds (Fig. S3, indicated by the green arrow), were excised and eluted for sequencing. The resultant sequences were then subjected to alignment analysis with the NCBI database using the online tool BLASTp, leading to the identification of 10 genes potentially related to enhancing MnODA. The function annotation of these genes is depicted in Fig. 1A.

To obtain MnODA-promoted mutant strains, the 10 identified target genes influencing MnODA activity were individually knocked out using the pDS3.0 homologous recombination technique (Fig. S4). The resulting 10 mutant strains were cross-checked through PCR amplifications using different primer combinations, which confirmed the successful knockout of the corresponding target genes in each mutant (Fig. S5). Subsequently, the Mn(II)-oxidizing activity of these mutant strains was compared with that of the wild-type MB04B. Figure 1B shows that after culturing under similar Mn(II)-inducible conditions for 3 days, the Mn(II)-oxidizing activity of MB04B was approximately 946 µmol/L. In contrast, nine mutant strains (MB04B△*00350*, MB04B△*10425*, MB04B△*15025*, MB04B△*09040*, MB04B△*10605*, MB04B△*18580*, MB04B△*19410*, MB04B△*19420*, and MB04B△*06795*) exhibited enhanced activity to varying extents. A maximum increase in Mn(II)-oxidizing activity by 25% ± 5% (*P* < 0.05) was recorded in the mutant MB04B△*06795*.

Due to the limited increase in MnODA observed after individual gene knockout, we performed further continuous progressive multiple knockouts of two to seven MnODA-related genes (three are associated with amino acid metabolism: *MYG11_19410, MYG11_09040,* and *MYG11_18580*; one encodes a diguanylate phosphodiesterase: *MYG11_00350*; one encodes a carboxyl terminal-processing peptidase (Ctp): *MYG11_06795*, one encodes a porin: *MYG11_10425*, and one encodes a gluconate operon repressor (GntR) family transcriptional regulator: *MYG11_15025*), using the mutant MB04B△*00350* as the parent strain. Consequently, an ultimate mutant strain with a complete knockout of all seven genes was generated, named MB04R-14. While the gradual knockout of two to seven genes resulted in corresponding MnODA enhancements in each intermediate mutant (Fig. S6), MB04R-14 exhibited the highest MnODA among all mutants tested, exceeding that of some previously reported bacteria (43, 44). Therefore, MB04R-14 was chosen as the parent strain for subsequent studies.

To compare the Mn(II)-oxidizing activities of the engineered strain MB04R-14 (undergone sequential knockout of seven genes) and the parent strain MB04B, both strains were cultured continuously for 168 h in liquid Lept medium supplemented with Mn(II). The MnODA was quantified at 12 h, 18 h, and 24 h intervals thereafter. As depicted in Fig. 1C, both strains exhibited a synchronous and steady increase in MnODA over the initial 120 h time course under normalized culturing conditions, after which both strains stabilized and maintained high levels until 168 h. However, MB04R-14 exhibited significantly quick Mn oxide formation and relatively higher MnODA profiles compared to the parent strain MB04B, with the highest increase in MnODA of approximately 35% recorded in 120 h, when both strains reached their peak activity. Furthermore, cultures of both strains grown on Lept plates also corroborated the faster Mn oxide formation of the engineered strain MB04R-14 over MB04B (Fig. 1D), as visualized by the gradually increasing and darkening colonies (indicative of Mn oxide formation on the cell surface) of MB04R-14 compared to mostly light yellow colonies of MB04B observed at 36 h, 60 h, 84 h, and 108 h. These results suggest that the engineered MB04R-14 not only exhibited increased Mn(II)-oxidizing activity but also accelerated the formation of Mn oxides compared to the parent strain MB04B.

### Characterization of BMO aggregates formed by MB04R-14

**Fig 2:**
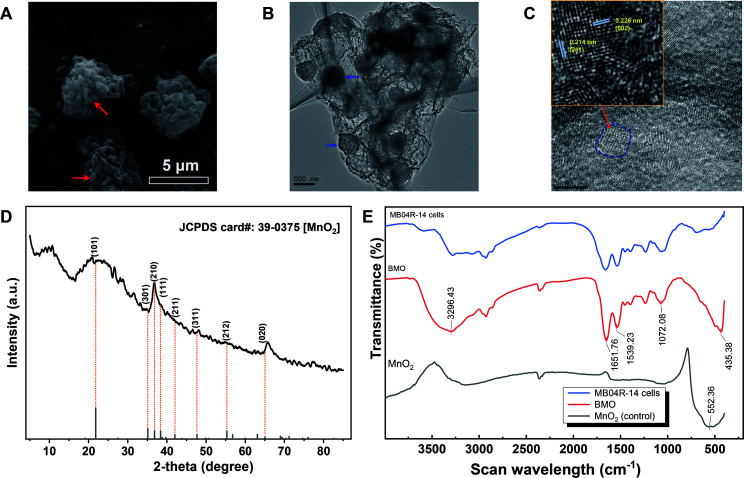
Structural characterization of BMO aggregates formed by MB04R-14. (**A**) SEM micrograph of the BMO aggregates; (**B**) typical HRTEM images of a randomly selected BMO aggregate; (**C**) measured lattice spacings of the micro/nanostructured BMO aggregate matter; (**D**) XRD pattern of Mn oxides of the BMO aggregates; (**E**) FTIR of the BMO aggregates.

After continuous 1 mmol/L Mn(II)-enriched culturing in liquid Lept medium for 48 h, the engineered strain MB04R-14 formed microspherical aggregates consisting of bacterial cells, Mn oxides, and organic matrix. Scanning electron microscopy (SEM) observation showed that these aggregates were irregular agglomerates with porous or hollow surfaces, approximately 5 µm–10 µm in diameter, and the presence of attached and embedded bacteria around the aggregates was easily discernible (Fig. 2A, indicated by red arrows). High-resolution transmission electron microscopy (HRTEM) analysis revealed these aggregates to consist of multiple compact particles dispersed within an interwoven network of organic matter fibers (Fig. 2B, indicated by blue arrows). The fine microstructure of individual particles exhibited distinct nanocrystalline configuration structures within the carbon matrix (Fig. 2C, indicated by the dashed circle). The lattice fringe values calculated as 0.214 nm and 0.226 nm corresponded to the *d-*spacing values of the (211) and (002) plane in ramsdellite-type MnO_2_, suggesting that the BMO aggregates constituted a micro/nanostructure MnO_2_ composite. Concurrently, X-ray diffraction (XRD) analysis of the aggregates revealed the characteristic diffraction peaks at 22°, 37°, 48°, and 65° corresponded to the (101), (210), (311), and (020) crystal planes of natural ramsdellite MnO_2_ (JCPDS card#: 39-0375) (Fig. 2D). This pattern also closely resembled those observed in other BMOs (30, 31).

The Fourier-transform infrared spectroscopy (FTIR) analysis (Fig. 2E) of the surface functional groups of BMOs revealed a new vibration peak at 435 cm^−1^ attributed to Mn–O bonding, along with two stronger and wider absorption peaks at 3,296 cm^−1^ and 1,651 cm^−1^, respectively, associated with H_2_O molecules, compared to lone MB04R-14 cells. Additionally, the Mn–O peak of BMOs exhibited a slight shift compared to that of chemically synthesized MnO_2_, suggesting a potential difference in Mn–O bond length as observed in both MnO_2_, as reported by Yashas et al. (45). These results collectively confirm that the BMO aggregates formed by MB04R-14 were micro/nanostructured composites primarily composed of ramsdellite (MnO_2_).

### Degradation of MTC and kinetic analysis by the BMO composite

**Fig 3:**
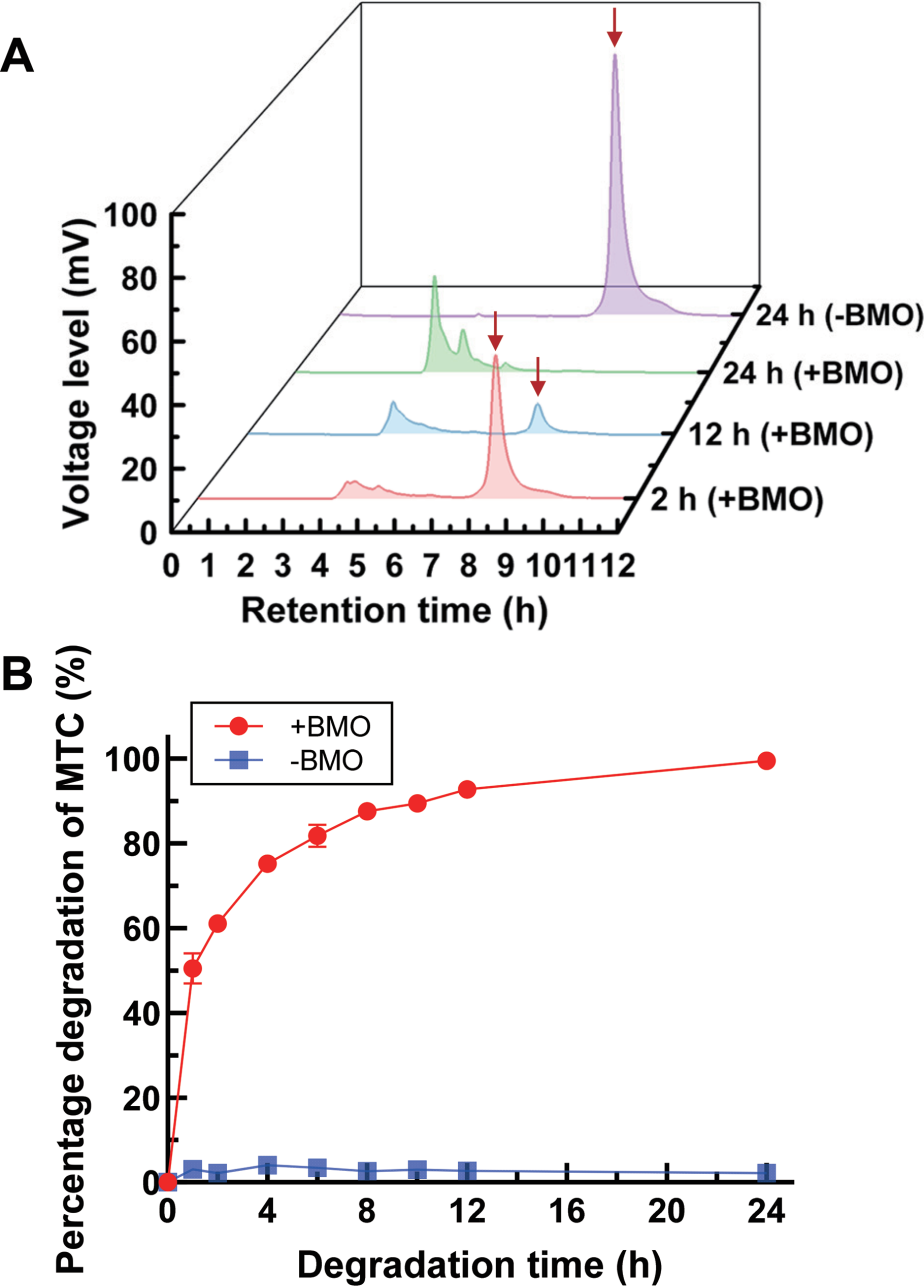
Degradation of MTC by the BMO composite. (**A**) HPLC chromatographs of elute fractions of MTC degradation in 2 h, 12 h, and 24 h, respectively. The MTC elution peak is indicated by the red arrow. (**B**) Degradation rate curve of MTC by the BMO composite. Error bars represent the standard error of the mean of three biological replicates. “+BMO” indicates the addition of BMO, while “-BMO” indicates the absence of BMO.

The degradation of MTC at an initial concentration of 50 µg/mL by the BMO composite produced from MB04R-14 was monitored using high-performance liquid chromatography (HPLC) assays over a 24 h reaction period. In Fig. 3A, the elution fractions of residual MTC (indicated by red arrows) in HPLC chromatograms at 2 h, 12 h, and 24 h post-reaction revealed rapid reduction in residual MTC levels. The degradation rates were approximately 61.0%, 92.9%, and 100% at 2 h (Fig. 3A, indicated by the red line), 12 h (Fig. 3A, indicated by the blue line), and 24 h (Fig. 3A, indicated by the green line), respectively, compared to no degradation observed in the 24 h negative control (Fig. 3A, indicated by the purple line). Quantitative analysis of MTC degradation also confirmed a steadily increasing degradation rate over the 24 h reaction time course, ultimately reaching 100% degradation at 24 h (Fig. 3B).

**Fig 4:**
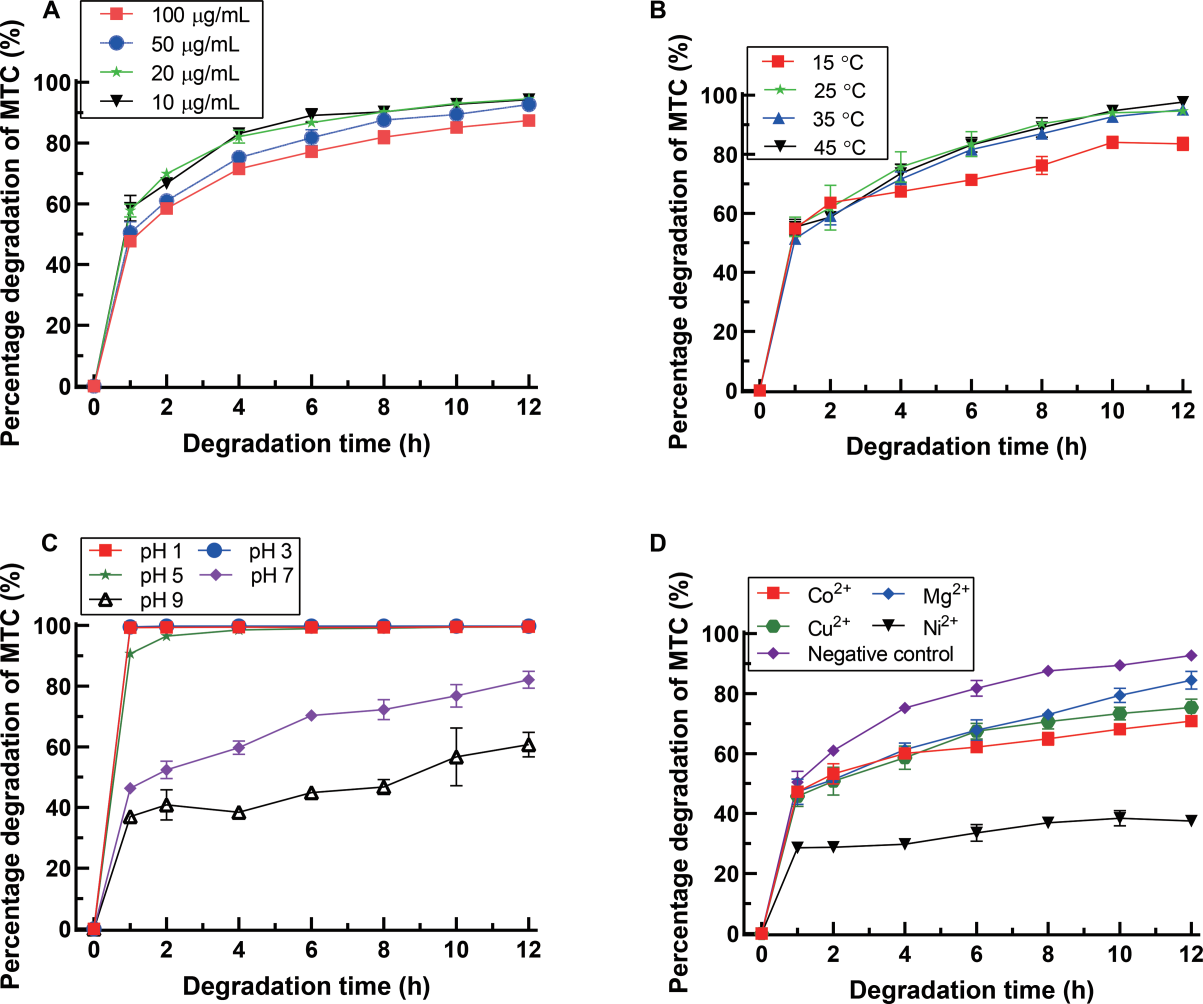
Effects of different initial MTC concentrations (**A**), temperatures (**B**), pH values (**C**), and metal ions (**D**) on MTC degradation by the BMO composites. The MTC concentration used in the experiments on temperature, pH, and metal ion effects is 50 mg/L. The negative control in panel D refers to the system containing only manganese oxides without the addition of other metal ions.

The impact of the initial concentration of MTC, temperature, pH, and various metal ions on the degradation efficiency of MTC by the BMO composite was examined. As depicted in Fig. 4A, escalating MTC concentrations from 10 μg/mL to 100 μg/mL resulted in a diminishing degradation rate from approximately 94% to 87%, indicating a dose-dependent correlation between BMO and MTC degradation. Figure 4B shows that lowering the temperature from 45°C to 15°C conducted a reduction in MTC degradation rate from approximately 97% to 83%, with an optimal moderate temperature range of 25°C to 45°C. Figure 4C illustrates the significant influence of pH on MTC degradation, with acidic conditions remarkably expediting the reaction rate. When the pH was below 3, 50 mg/L of MTC was completely degraded within 1 h. Whereas, at pH 5, approximately 90% degradation was achieved within the same time frame, with degradation efficiency significantly declining as the pH increased further. Figure 4D showed that metal ions (Mg^2+^, Cu^2+^, Ni^2+^, and Co^2+^) exhibited varying degrees of inhibitory effects on MTC degradation, with Ni^2+^ leading to a maximum reduction of 55% in MTC degradation rate compared to other metal ions.

**Fig 5:**
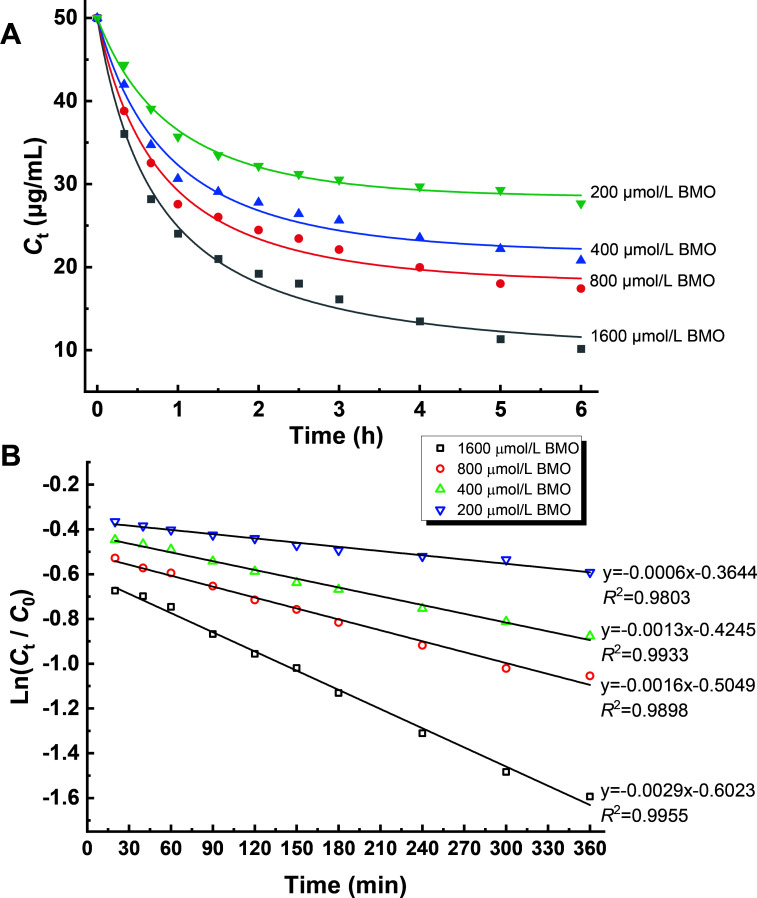
Kinetics analysis of MTC degradation by BMO. (**A**) Degradation reaction time course with different initial BMO concentrations; (**B**) the plotted linear models of degradation kinetics.

MTC degradation kinetics experiments were conducted to analyze the first 6 h degradation data of the BMO composite at different concentrations. As shown in Fig. 5A, synchronized degradation curves across all treatments show rapid degradation within the initial 2 h, followed by relatively stable degradation thereafter. Figure 5B shows that the fitted data exhibit good consistency with the pseudo-first-order degradation reaction kinetic model (equation 2), with all treatments showing linear fits and *R*^2^ values exceeding 0.98, and the rate constant (*k*) values increased from 0.0006 to 0.0029 as the concentration of the BMO composite increased from 200 μmol/L to 1600 μmol/L, indicating that the degradation of MTC was highly dependent on the BMO composite, highlighting its crucial role in MTC degradation.

### Constructive degradation-metabolic pathway

**Fig 6:**
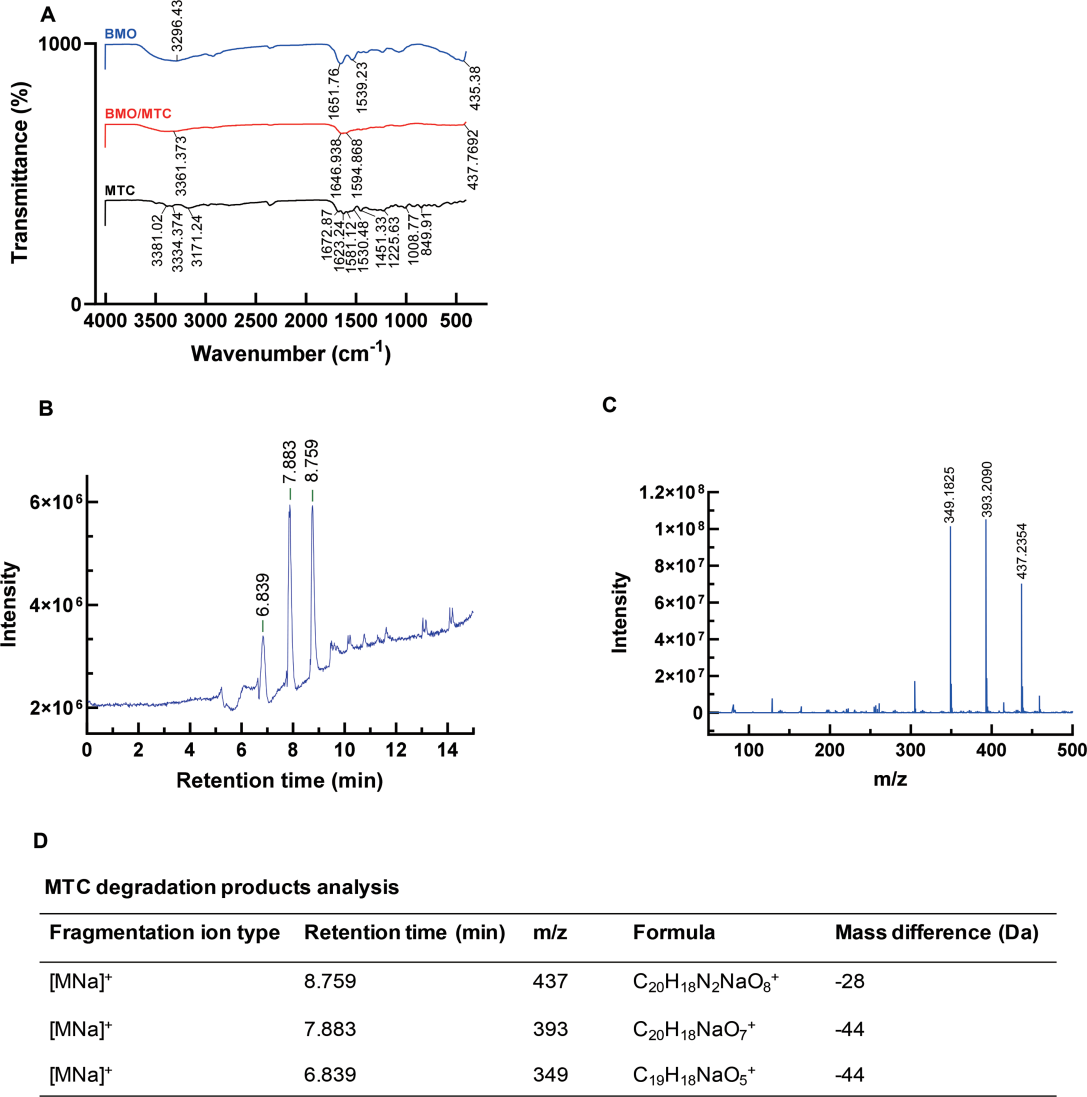
FTIR analysis of BMO after 24 h degradation of MTC (**A**), Liquid chromatography-mass spectrometry analysis of the eluted peaks of MTC-degraded intermediates during BMO degradation (**B**), the scanning curve of three main intermediates by TIC (**C**), and predicted formula of three main intermediates (**D**).

To further identify structural groups of BMO involved in the MTC degradation, the FTIR spectrum of the reacted BMO after a 24 h degradation period was compared with that of unreacted BMO and lone MTC. In Fig. 6A, a significantly weakened vibration peak of Mn–O in the reacted BMO at 435 cm^−1^ after reacting with MTC indicates the consumption of BMO during the reaction. Additionally, the spectrum of the reacted BMO showed broadening in the range of 1,480–1,800 cm^−1^, along with a new absorption peak at 1,594 cm^−1^; and a reduction and shift in the hydroxyl absorption peak at 3,296 cm^−1^. These changes are associated with characteristic peaks of MTC. In the MTC spectrum, peaks at 1,672 cm^−1^ and 1,530 cm^−1^ are attributed to the carbonyl group and the amino group on the A-ring, respectively. Peaks at 1,623 cm^−1^ and 1,581 cm^−1^ correspond to the carbonyl groups on the A-ring and C-ring, respectively. The peak at 1,451 cm^−1^ is associated with the C = C skeleton, and the peak at 3,381 cm^−1^ corresponds to the hydroxyl group. These observed post-degradation changes in BMO suggest that MTC was likely adsorbed initially onto the surface of the BMO composite, as noted in previous studies (46, 47), and subsequently underwent complete degradation by the BMO composite.

**Fig 7:**
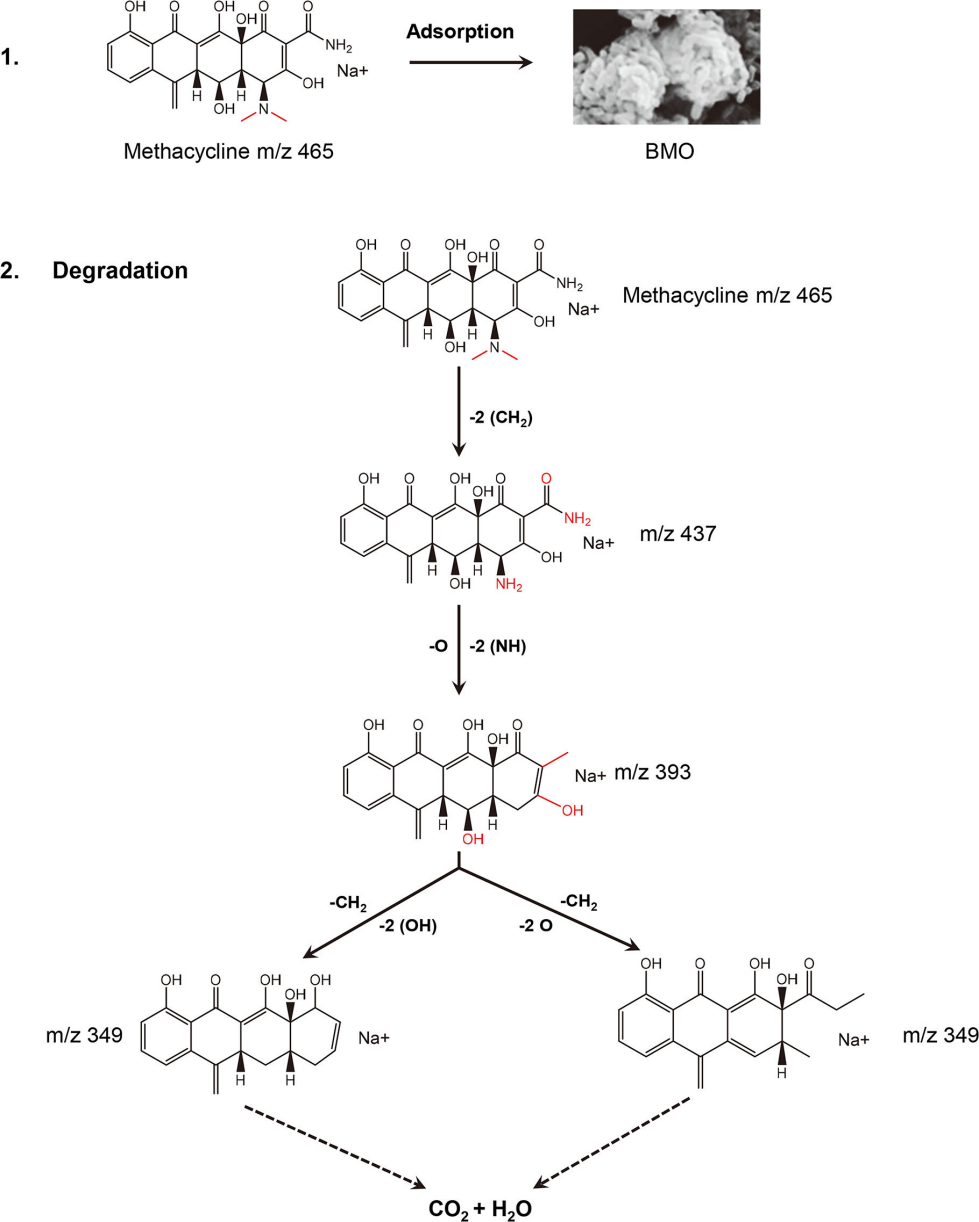
Constructive degradation-metabolic pathway of MTC by the BMO composite.

The liquid chromatography-mass spectrometry (LC-MS) analysis revealed three intermediate products of MTC degradation by the BMO composite, with retention times of 8.759, 7.883, and 6.839 min (Fig. 6B). These intermediate molecules showed m/z values of 437, 393, and 349, respectively (Fig. 6C), corresponding to substances with predicted molecular formulas (Fig. 6D) based on a highly matched search in the chemical database. Nevertheless, the occurrence of these intermediates not only demonstrates that the BMO composite was efficient in eliminating MTC but also indicates that the removal of MTC by the BMO composite occurred through degradation rather than mere adsorption. Based on previous reports (34, 48) and the LC-MS assay patterns of MTC-degraded products, we propose a metabolic pathway for the degradation of MTC by the BMO composites as illustrated in Fig. 7. Initially, MTC undergoes a process where two amino-methyl groups are eliminated from the A-ring, resulting in an intermediate product with an m/z of 437. Subsequently, the A-ring undergoes the removal of both the amino and amide groups, leading to the formation of an intermediate product with an m/z of 393. Further steps involve the removal of one hydroxyl group from each of the A- and B- rings, followed by the loss of a methyl group from the A-ring, resulting in an intermediate product with an m/z of 349. Eventually, complete degradation occurs. Due to the relatively rapid degradation of MTC by BMO and the late sampling time, fewer identified products were detected; hence, some other intermediates may remain unidentified.

### Antibacterial activity of MTC-degraded products

**Fig 8:**
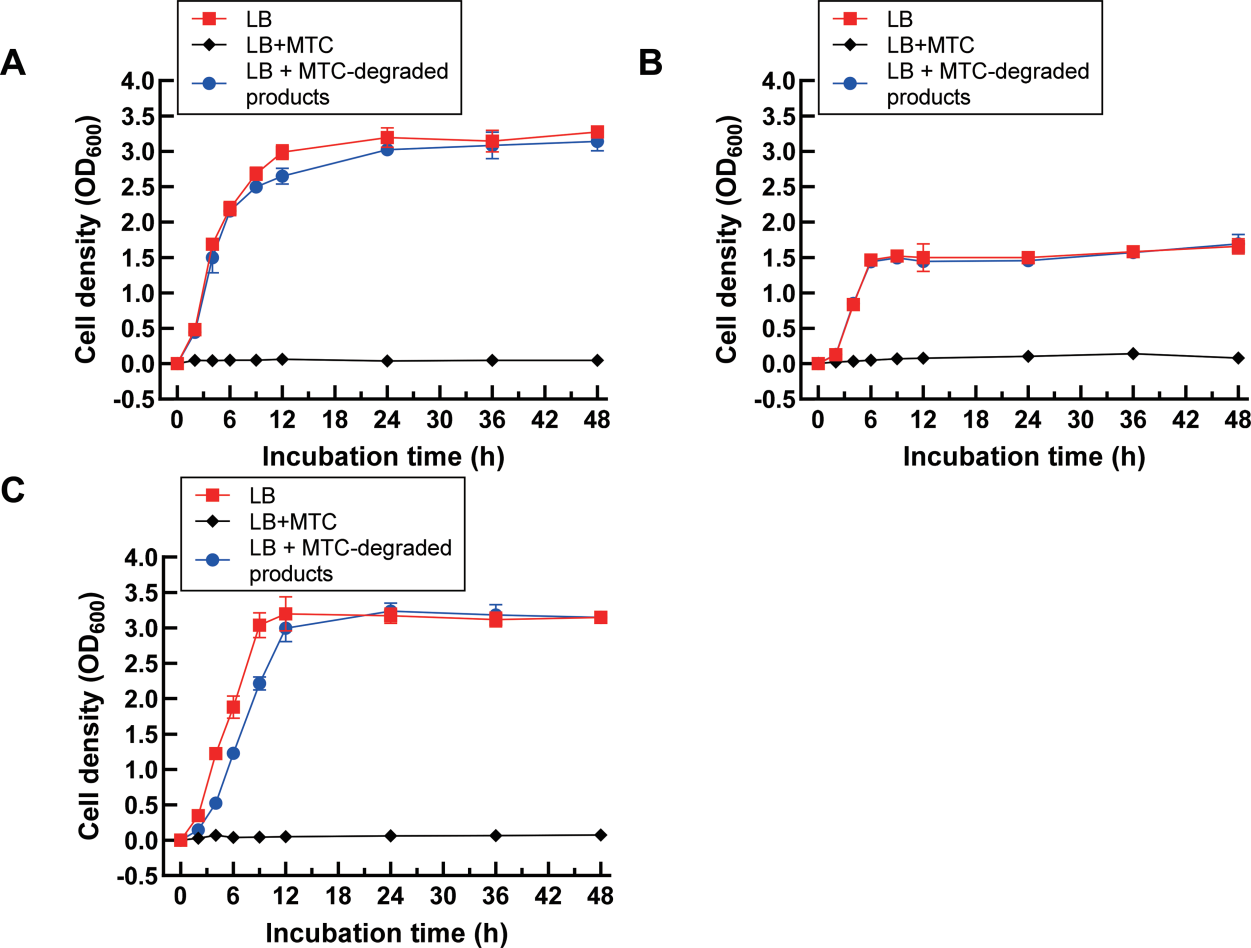
Growth effect of MTC-degraded products on *P. aeruginosa* ATCC15442 (**A**), *E. coli* DH5α (**B**), and *S. aureus* KCTC1621 (C) over a 48 h culture time course.

To assess whether the 24 h degraded product of an initial 50 µg/mL concentration of MTC by the BMO composite retains its antibiotic activity, we measured the minimum inhibitory concentration (MIC) of MTC against *Escherichia coli* DH5, *Pseudomonas aeruginosa* ATCC15442, and *Staphylococcus aureus* KCTC1621 (in the ranges of 3.125 μg/mL–6.250 μg/mL, 1.5625 μg/mL–0.7813 μg/mL, and 0.0977 μg/mL–0.0489 μg/mL, respectively) (Fig. S7A). The inhibition zone test of the MTC degradation product solution was then performed on Luria–Bertani (LB) agar plates inoculated with these three bacterial strains. Figure S7B shows that there are no visible inhibition zones surrounding the Oxford cups loaded with undiluted, double-diluted, and triple-diluted MTC-degraded product solutions. This starkly contrasts with the pronounced inhibitory zone observed for untreated MTC. To quantitatively evaluate the antibacterial activity of the degradation products of MTC, *E. coli* DH5, *P. aeruginosa* ATCC15442, and *S. aureus* KCTC1621 were utilized as representative gram-negative and gram-positive bacteria, respectively. As depicted in Fig. 8, no discernible growth inhibition was observed for gram-negative *P. aeruginosa* ATCC15442 (Fig. 8A) and *E. coli* DH5 (Fig. 8B) and gram-positive *S. aureus* KCTC1621 (Fig. 8C) when exposed to the MTC-degraded product, suggesting that following treatment with the BMO composite, the antibacterial activity of MTC was eliminated.

### MTC degradation on MTC-spiked hospital wastewater and continuous degradation

**Fig 9:**
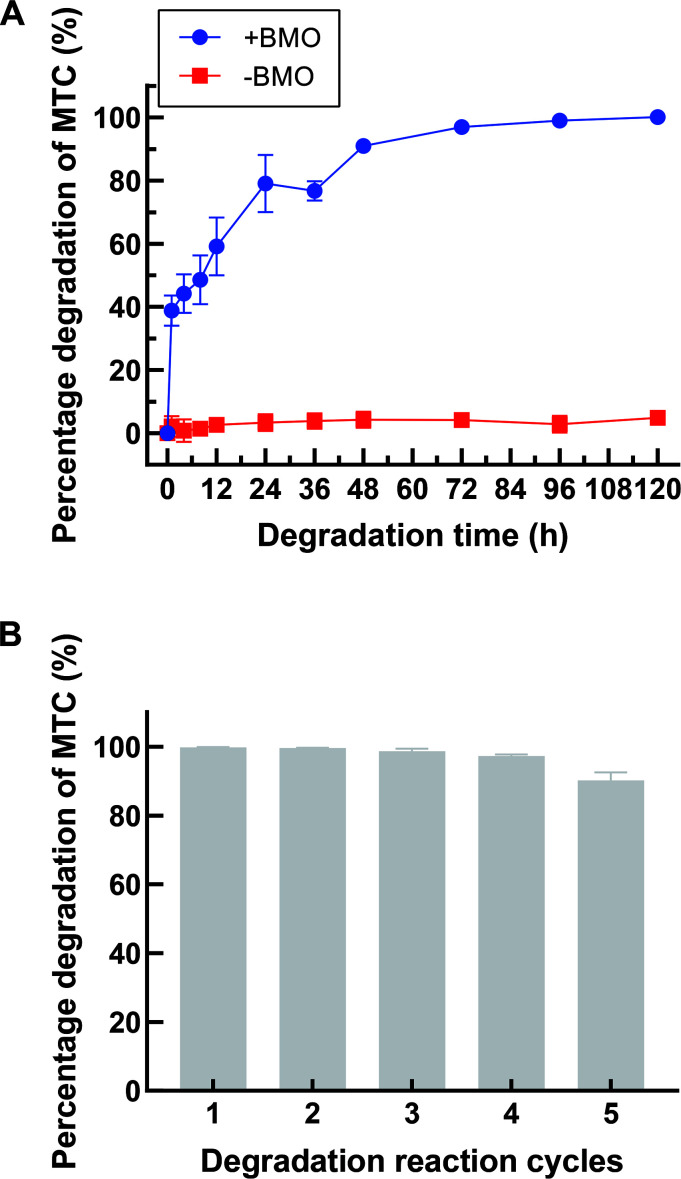
Degradation time course of MTC in MTC-spiked hospital wastewater (**A**), and continuous five-round degradation of MTC (**B**) by the BMO composites. “+BMO” indicates the addition of BMO, while “-BMO” indicates the absence of BMO.

To evaluate the practical utility of the prepared BMO composite for treating naturally MTC-contaminated wastewater, hospital wastewater (pH approximately 7.4) was spiked with 50 µg/mL MTC to the final concentration. As depicted in Fig. 9A, approximately 99% of the MTC was degraded within 3 days. In comparison, less than 5% of the MTC was degraded in the MTC-containing wastewater without adding BMO during a similar time course. While the degradation efficiency appears to be slower under real wastewater conditions compared to laboratory trials using dH_2_O (with a pH around 6.7), the significant degradation activity of the BMO composite in real wastewater suggests its potential applicability. Furthermore, the recyclability and repeated performance of the BMO in treating MTC were assessed. As shown in Fig. 9B, the BMO composites maintained high MTC degradation activity over five rounds, with degradation rates of 99.8%, 99.6%, 98.7%, 97.3%, and 90.2% in rounds 1–5, respectively. These results indicate the serially reusable performance of the BMO composites in degrading MTC.

## DISCUSSION

Recently, we reported the crucial role of metabolic pathways and specific carbon metabolism-related genes in Mn oxide formation by a soilborne Mn(II)-oxidizing bacterium (49). The current study demonstrated that the simultaneous knockout of seven genes in the engineered strain MB04R-14 resulted in a significant increase in MnODA and pronounced Mn oxide formation, indicating the profound involvement of these genes in Mn(II)-oxidizing activity within the host cells. Functional annotation of these genes revealed intriguing insights into their functions. This diverse functionality hints at the presence of complex and multilayered networks and mechanisms underlying Mn oxide formation, facilitating cellular adaptation to various conditions, including harsh environments (50). Although the detailed mechanisms remain elusive, based on our studies and previous reports, we speculate that the significant modulation of MnODA in MB04B with the knockout of these genes could occur primarily through indirect modulation of biofilm formation (e.g., *MYG11_00350, MYG11_09040,* and *MYG11_15025*), which may affect the levels of intracellular second messenger c-di-GMP. Additionally, certain genes may directly impact the structural integrity of the cell membrane (e.g., *MYG11_06795* and *MYG11_10425*), thereby ultimately affecting MnODA.

In the genus *Pseudomonas*, biofilm formation is predominantly regulated by c-di-GMP, a ubiquitous signaling molecule that governs the shift of bacteria from a free-floating lifestyle to an attached one (51). It is well-recognized that increased cellular levels of c-di-GMP bolster biofilm formation, whereas reduced levels of c-di-GMP favor bacterial motility and facilitate biofilm dispersal (52). Cellular c-di-GMP is typically synthesized through the catalysis of a diguanylate cyclase containing a GGDEF domain from two molecules of GTP and hydrolyzed by an EAL domain- or HD-GYP domain-containing phosphodiesterase (PDE) into 5′-phosphoguanylyl-(3′,5′)-guanosine (53, 54). The protein encoded by *MYG11_00350* harbors an EAL domain, a pivotal structural domain facilitating specific PDE activity targeting c-di-GMP. Upon the knockout of *MYG11_00350*, the degradation of intracellular c-di-GMP in MB04R-14 diminishes, leading to increased accumulation of c-di-GMP and, consequently, augmented biofilm production (Fig. S8). A previous study has reported that biofilm matrices encompass regions with both positive and negative charges. These sites potentially aid in the adsorption of ions by the biofilm via electrostatic interactions and ion exchange in the adjacent aqueous environment, which enables microorganisms within the biofilms to readily utilize these ions as nutrient ions (55). It is hypothesized that the increased intracellular levels of c-di-GMP in MB04R-14 will accelerate cell aggregation, thereby fostering biofilm proliferation. The escalated biofilm formation enhances the absorption and transport of Mn^2+^, resulting in increased MnODA levels and an accelerated rate of BMO formation. Furthermore, some investigations also suggest that c-di-GMP mediates the upregulation of Mn(II) oxidation activity in *Pseudomonas resinovorans* by inducing the expression of manganese peroxidase (56).

Several previous investigations have documented the crucial role of specific amino acids in regulating biofilm formation in different bacteria. For instance, in *Pseudomonas aeruginosa*, it has been observed that an increased concentration of arginine stimulated surface adhesion (57). These phenotypic changes can be attributed to variations in the intracellular levels of c-di-GMP. Additionally, certain amino acids such as tryptophan, which positively influence c-di-GMP, have been identified, and a synergistic effect between arginine and tryptophan has been observed (58). In another investigation on *P. aeruginosa*, it was revealed that arginine not only acts as an environmental signal but also serves as a metabolic indicator. Mutations affecting arginine transport or biosynthesis have been found to cause alterations in c-di-GMP levels and biofilm formation (59). Similarly, in *Salmonella*, arginine has been reported to regulate the level of c-di-GMP (60). The *MYG11_09040* gene encodes arginine succinyltransferase, a crucial enzyme involved in the bacterial arginine metabolism pathway. Thus, we hypothesize that knockout of this gene disrupts the arginine succinyltransferase pathway, leading to an accumulation of arginine. Consequently, it is likely to further result in increased biofilm formation and enhanced MnODA production in MB04R-14. Moreover, the GntR transcription factor family represents a large group of regulatory proteins distributed in various bacterial species, which regulate the expression of diverse genes encoding ABC transporters, membrane proteins, extracellular enzymes, and factors involved in biofilm formation. Previous studies have demonstrated the significant enhancement of biofilm formation by a GntR family regulator, MpaR, in *P. aeruginosa* (61), and other GntR family regulators in different bacteria such as *Vibrio parahaemolyticus* (62), *Listeria monocytogenes* (63), and *Lysobacter capsici* (64), among others. Hence, it is conceivable that the disruption of *MYG11_15025* contributes to the increased biofilm formation and MnODA production in the engineered MB04R-14.

The protein encoded by *MYG11_06795*, Ctp, is a serine protease involved in multiple cellular processes through the post-translational modification of proteins. A previous investigation in *Burkholderia baumannii* demonstrated that disrupting Ctp function enhances biofilm formation (65). Moreover, Ctp proteins in gram-negative bacteria are commonly situated in the periplasmic compartment and have been implicated in cleaving proteins associated with transmembrane and cell envelope functions (66). For instance, in *P. aeruginosa*, CtpA forms a complex with LbcA, regulating several peptidoglycan cross-linking hydrolases, thereby altering cell membrane structure and influencing the type III secretion system (67). Considering that bacterial BMO is typically formed on the cell surface, we speculate that alterations in cell membrane structure by certain gene disruptions may impact the Mn oxidation process and alter Mn(II)-oxidizing activity.

The degradation efficiency of MTC by BMO was significantly affected by the initial MTC concentration, temperature, and pH (see Fig. 4). It is noted that the surface reaction kinetics model proposed by reference 68 may be applicable in describing the degradation reaction of MTC by BMO. During redox reactions, an initial precursor complex forms between organic substrates and δ-MnO_2_, followed by electron transfer. These two steps are regarded as rate-limiting and are the primary factors influencing the reaction between organic substrates and BMO. Consequently, the extent of contact between the organic substrates and BMO dictates the oxidation rate. Excessive MTC can lead to competition among reaction sites. When the concentration of MTC exceeds the available surface sites of BMO within the system, these sites become saturated with the organic substrates. The introduction of additional organic reducing agents fails to increase the formation of precursor complexes, leading to a reduction in the reaction rate and a decline in degradation efficiency (69). Moreover, as previously noted, the initial stage of MTC degradation by BMO involves the adsorption of MTC onto the surface of BMO to form precursor complexes. Elevated temperatures enhance the mass transfer rate of MTC from the solution to the BMO surface. Consequently, the degradation efficiency was improved with increasing temperature. Theoretically, the pH value can affect the surface charge and dissociation of functional groups on adsorbent active sites, as well as the chemical speciation of substrates in the solution. Studies indicate that lowering the solution pH can increase the presence of surface substances conducive to interaction with reducing agents. It has been reported that pH not only affects affinity but also modulates the redox potential of reactants (70). Lower pH values not only increase the reduction potential of oxidants but also promote the protonation of precursor complexes, thereby facilitating electron transfer reactions. The weakly crystalline Mn oxides present in BMO contain plenty of cationic vacancies. Coupled with their irregular and naturally extensive surface area, BMO aggregates promote the adsorption and entrapment of cations (such as Mg^2+^, Cu^2+^, Ni^2+^, and Co^2+^) at the BMO interface. This occupation of reaction surface sites hinders electron transfer and competes with organic pollutants for reaction sites, resulting in a decrease in degradation efficiency (36).

It is noteworthy that the BMO prepared in this study displays promising potential for efficiently treating low-pH organic pollutants. Various industries, including ammunition, pharmaceuticals, mining, steel production, electroplating, and phosphorus processing, discharge highly acidic effluents, which pose significant environmental challenges due to their high toxicity and carcinogenicity. The acidity of such wastewater results in the extensive dissolution of heavy metals and other pollutants, inhibiting microbial growth. The BMO material developed in this study exhibits promising performance under acidic conditions, demonstrating remarkable degradation efficiency for MTC across the pH range of 1 to 5 (see Fig. 4). Furthermore, its reusability in repeated performance and effectiveness in treating actual MTC-containing hospital wastewater suggest its potential for further investigation in large-scale or continuous degradation processes.

In summary, an Mn(II)-oxidizing bacterium was genetically modified in the current study to prepare a micro/nanostructured BMO composite that enables complete degradation of the polycyclic antibiotic MTC. A mutagenesis library of MB04B was initially constructed using the mini-Tn5 transposon mutation system, and 10 genes with enhanced Mn oxidation activity were identified using hiTAIL-PCR technology. By simultaneous knockout of seven genes, the mutant strain MB04R-14, with a 35% increase in MnODA and accelerated BMO formation, was yielded. The multiple-phase composition and fine structure of BMOs were characterized using SEM, HRTEM, FTIR, and XRD. HPLC and LC/MS analysis confirmed the complete degradation of MTC. The degradation of MTC by BMO can be conducted over a wide range of pH (1–5) and temperature (15°C–45°C), with good continuous performance and a good capacity to detoxify real hospital wastewater containing MTC. Residual antibacterial bioassays on several indicative bacteria verified the complete elimination of antibiotic activity from the degradation products.

## MATERIALS AND METHODS

### Reagents

Hydrochloride MTC (purity ≥ 98%) was procured from Shanghai Macklin Biochemical Technology Co., Ltd. (Shanghai, China). Methanol (purity ≥ 99.8%) was purchased from Beijing MREDA Science and Technology Co., Ltd. (Beijing, China). MTC was initially dissolved in an appropriate volume of ethanol and subsequently diluted to a stock solution of 10 mg/mL using deionized water. The stock solution was then filtered through a sterile 0.22 µm microfiltration membrane before use. All other chemicals used were of analytical grade or higher.

### Bacterial strains, plasmids, and bacterial culture

The bacterial strains used in this study are listed in Table S1. Specifically, the wild-type strain *Pseudomonas* sp. GSICC31629 (Gansu Culture Collection Center, Lanzhou, China), designated MB04B, with significant Mn(II)-oxidizing activity (41), served as the parental strain for various genetic manipulations, including transposon mutagenesis library construction, gene-knock mutant generation, and the preparation of the BMO aggregate composite for MTC-degradation experiments. *Escherichia coli* DH5α was used as a recipient strain for constructing different chimeric genes and conducting related recombinant experiments, while *E. coli* S17-1/λ pir was the recipient strain for constructing a mini-Tn7 insertion plasmid vector. The gram-positive wild-type strain *Staphylococcus aureus* KCTC1621 and the gram-negative strains *Pseudomonas aeruginosa* ATCC15442 and *Escherichia coli* DH5α were used as bacterial indicators for bioassays of growth inhibition by MTC-degraded products.

All bacterial strains were routinely cultured in LB medium (71), with *Pseudomonas* strains incubated at 28°C and other strains at 37°C. An MG medium (1% mannitol, 0.2% L-glutamic acid sodium, 0.05% KH_2_PO_4_, 0.02% NaCl, 0.02% MgSO_4_) was used for transposon mutagenesis library construction. For inducing the synthesis of BMO aggregate composites, MB04B or its derivative strains were cultured in Lept medium (72) containing 1 mmol/L MnCl_2_ at 28°C with agitation at 150 rpm. A 10× Vogel-Bonner minimal medium (VBMM) stock solution and VBMM agar medium were prepared according to a previously described method (73). Ampicillin (Amp) or kanamycin (Kan) was added to LB or Lept medium at final concentrations of 100 µg/mL and 50 µg/mL, respectively, for culturing recombinant *E. coli* and *Pseudomonas* cells, as needed.

### Mn(II)-oxidizing activity and MnODA determination

Mn(II)-oxidizing activity of whole-cell suspensions was determined using the standard LBB method (74) based on the quantification of Mn oxides (primarily assumed to be MnO_2_). To measure the absorbance at 620 nm, KMnO_4_ was used as a standard. The concentration of MnO_2_ produced (1 mmol/L MnO_2_ corresponds to 0.4 mmol/L KMnO_4_) was defined as the concentration of Mn oxides, which refers to the MnODA value of cell suspension.

### Construction of mutation library and identification of transposition insertion sites

The construction of the mini-Tn5 transposon mutagenesis library for the Mn(II)-oxidizing *Pseudomonas* MB04B and screening target genes corresponding to MnODA promotion is outlined in Fig. S1. In brief, an overnight culture mixture containing the donor strain (*E. coli* S17-λpir carrying the transposon plasmid puT-mini Tn5-Km2) and the recipient strain (MB04B) in a 1:2 ratio was suspended in a 10 mM MgSO_4_ solution, was then dropped onto sterile filter paper and placed on LB plates. Following incubation at 28°C for 24 h, cells were washed off the filter paper with phosphate buffered saline (PBS), spread onto Kan^r^ MG plates, and further incubated at 28°C for an additional 36 h. Colonies that exclusively grew on Kan^r^ plates, but not on Amp^r^ plates, were considered successful transposition mutants. The identification of transposon insertion sites in the selected transposition mutants was conducted using the hiTAIL-PCR method (75). After three rounds of PCR amplification and agarose gel electrophoresis of the amplified products, sequencing was performed on the excised bands from the third amplification round, which were 100 bp–200 bp smaller than those from the second round. Subsequently, alignment analysis was carried out using the genomic data of MB04B (GenBank accession no. JALLPP000000000) to pinpoint the target genes responsible for the MnODA promotion.

### Gene knockout

The plasmids and PCR primers used in this study are listed in Tables S2 and S3, respectively. The technical procedures for gene knockout in MB04B are illustrated in Fig. S4. Briefly, based on the sequences of the identified 10 target genes associated with increased MnODA (Fig. 1A), flanking arms of approximately 1,000 bp each were amplified from the genomic DNA of MB04B. Subsequently, these fragments were seamlessly ligated with the *Sac*I-digested plasmid pDS 3.0 using the Gibson assembly method (76). The knockout vectors thus constructed were successfully introduced into MB04B via conjugation with *E. coli* S17-1/λ pir, and the transformed colonies were selected on VBMM solid medium supplemented with Kan. Colonies exhibiting slower growth on VBMM solid medium containing 15% sucrose were selected. After overnight cultivation, these colonies were diluted and spread on VBMM plates with 15% sucrose. Colonies that did not grow on the Kan^r^ medium were further confirmed through cross-PCR amplifications of the products, followed by sequencing to demonstrate the successful knockout of the target genes. A similar strategy was employed to construct the mutant strain MB04R-14 with seven genes knocked out. Each subsequent gene knockout was executed based on the knockout of the preceding gene, continuing until all seven genes were successfully knocked out. Identification of these intermediate mutant strains and MB04R-14 was carried out through cross-PCR amplifications of the products followed by sequencing.

### Preparation of the BMO aggregate composite

The MB04R-14 cells cultured overnight were inoculated at a 2% (vol/vol) inoculum size into 200 mL of Lept medium containing 2 mM Mn^2+^, followed by incubation at 28°C with shaking at 150 rpm for 7 days. The suspended culture was then harvested, and the BMO aggregates were washed three times with dH_2_O (with a pH around 6.7). For MTC degradation, unless otherwise specified, the prepared composite was dissolved in dH_2_O (with a pH around 6.7) to a final concentration of 1.6 mM MnODA in the degradation reaction solution.

### Electron microscopy, XRD, and FTIR

MB04R-14 was cultured in 1 mM MnCl_2_ for 48 h, and the BMO aggregate material was freeze-dried to yield a powder composite, followed by assays of fine structure and element composition of the formed BMO aggregate composite using SEM (JSM-6390/LV, JEOL, Japan) and HRTEM (JEM-2100F, JEOL, Tokyo, Japan) equipped with an energy-dispersive spectroscopy detector, as previously described (77). The samples were characterized using a Rigaku Ultima IV (Japan) XRD, and a VERTEX 70 (Bruker, Germany) FTIR, following methods described previously (41, 77).

### Degradation of MTC

The degradation experiments were conducted in 50 mL Erlenmeyer flasks with a total reaction volume of 20 mL unless otherwise specified. These flasks were covered with tinfoil to maintain darkness. The reaction system consisted of BMO (1.6 mM) and MTC (initially at a concentration of 50 mg/L) dissolved in dH_2_O (with a pH around 6.7). The mixed system was then incubated on a rotary shaker at 28°C with constant agitation of 160 r/min. In parallel, MB04R-14 was used as a control instead of BMO for the MTC degradation. Each experiment was carried out with at least three replicates for statistical robustness.

During certain time intervals of the MTC degradation time course, the MTC content was determined using a Shimadzu LC-20A HPLC system (Shimadzu, Kyoto, Japan), equipped with a Hypersil ODS2 chromatographic column (5 μm × 250 mm × 4.6 mm). The mobile phase consisted of 50% water (pH adjusted to approximately 3 with H_3_PO_4_) and 50% methanol, with a flow rate of 0.7 mL/min. Detection wavelengths were set at 280 nm and 350 nm, and the column temperature was maintained at room temperature for isocratic elution, following the previously described procedures (78). Before the HPLC assay, the MTC-degradative reaction solutions were mixed with an equal volume of 5 mg/mL oxalic acid to stop the reaction (79). After centrifugation at 10,000 rpm for 10 min, the supernatant was collected and filtered through a 0.22 µm organic filter membrane to remove insoluble substances. The degradation rate was calculated using the following formula:


(1) $$Degradation\;rate\;(\%)=\frac{C_{0}-C_{t}}{C_{0}}\;\times 100\%.$$

where *C_0_* denotes the initial MTC concentration, and *C_t_* denotes the determined MTC concentration at time *t* in the degradation system.

To identify intermediate metabolites, the 24 h degradation products were subjected to centrifugation at 10,000 rpm for 10 min to collect the supernatant. The precipitate was washed three times with dH_2_O (with a pH around 6.7), dissolved in a NaOH solution (pH 9.0), and the supernatants were then combined after centrifugation. The resulting supernatant was filtered through a 0.22 µm filter membrane and analyzed using HPLC equipped with triple quadrupole mass spectrometry (LC-MS/QQQ), specifically an Agilent 1200/6460 LC/QQQ system (6460, Agilent, CA, USA) with a Waters Acquity BEH C18 column (2.1 mm × 50 mm × 1.7 µm). The mobile phase consisted of a methanol-NaOH aqueous solution (pH 9.0), with a flow rate of 0.4 mL/min. Detection was performed using an electrospray ion source in positive ion mode.

The optimization experiments of MTC degradation were conducted to investigate the factors affecting the MTC degradation efficiency by the prepared BMO, including the initial MTC concentrations at the range of 10 μg/mL to 100 μg/mL, temperatures ranging from 15°C to 45°C, pH values from 1 to 9 (adjusted with phosphoric acid and triethylamine), and the addition of certain metal ions (Co^2+^, Mg^2+^, Cu^2+^, and Ni^2+^) at a concentration of 1 mmol/L. The MTC concentration used in the experiments on temperature, pH, and metal ion effects is 50 mg/L.

### MTC degradation reaction kinetics

The degradation reaction kinetics of the BMO composite across initial concentrations of 200 µmol/L, 400 µmol/L, 800 μmol/L, and 1,600 μmol/L toward the initial 50 µg/mL MTC was determined. The residual MTC contents in the reaction solutions were determined via HPLC assays after reaching degradation reaction equilibrium. The data from the first 6 h degradations were applied to plot degradation kinetics curves for the reaction using the following Equation:


(2) $$ln\frac{C_{t}}{C_{0}}=-kt.$$

where *C_0_* is the initial content (μmol/L) of MTC, *C_t_* is the content of residual MTC (μmol/L) at time *t* (min), *k* is the degradation rate constant, and *t* is the degradation reaction time (min).

### Detection of antibacterial activity of MTC degradation products

Following determining the antibacterial MIC of MTC against *E. coli* DH5α, *S. aureus* KCTC1621, and *P. aeruginosa* ATCC15442 according to a double-dilution method (80), MTC degradation products were assayed for the residual antibacterial activity against *E. coli* DH5α, *S. aureus* KCTC1621, and *P. aeruginosa* ATCC15442 by measuring the inhibition zones on LB agar plates. A suitable amount of *E. coli* DH5α, *S. aureus* KCTC1621, and *P. aeruginosa* ATCC15442 cells was evenly spread on a freshly prepared LB plate. Four sterile Oxford cups were then evenly placed on the agar surface. Under aseptic conditions, the MTC degradation product solution was filtered through a 0.22 µm microporous membrane, and an equal volume of the filtrate was poured into each cup. After incubation at 37°C for 24 h, the diameter of the inhibition zones was measured using a vernier caliper.

The antibacterial activity of MTC degradation products was further quantified using gram-negative *P. aeruginosa* and *E. coli* DH5α and gram-positive *S. aureus* as indicator strains. After 24 h degradation reaction on 100 mg/L MTC with the BMO composite, the degradation solution, untreated 100 mg/L MTC (serving as positive control), and sterile dH_2_O (as the negative control, with a pH around 6.7), each of these solutions was mixed with equal volumes of LB broth and equal volumes of each indicator strain. The mixtures were incubated at 37°C with shaking at 150 rpm for 72 h. The growth curve of each cell suspension was monitored by determining the optical density at 600 nm that was recorded and calculated based on the dilution multiples.

### Degradation of MTC-spiked hospital wastewater

The hospital wastewater (pH approximately 7.4) was physically filtered to remove large particulate impurities, and MTC (purity ≥ 98%) was subsequently added to the wastewater samples to a final concentration of 5 µg/mL. Then an appropriate amount of BMO composite was added to the reaction solution, and the reactions were allowed to proceed for 5 days. The remaining content of MTC in the reaction solutions was determined at each 12 h interval throughout the degradation time course.

### Continuous and repeated degradation performance

Continuous degradation experiments were conducted in 50 mL Erlenmeyer flasks, which were wrapped with tinfoil to prevent light interference. Each flask contained 20 mL of reaction solution consisting of MTC at an initial concentration of 50 µg/mL and 1.6 mM BMO composite. Following the degradation reaction for 24 h at 28°C with constant agitation at 160 rpm, the residual MTC content was determined, and the degradation rate was calculated using equation (1). After each cycle of the degradation reaction, the BMO composites were harvested by centrifugation, washed three times with dH_2_O (with a pH around 6.7), and used directly for the next cycle of degradation in MTC-containing solution, similar to the first round of reaction.

### Data analysis

All data are presented as the mean ± standard deviation of at least three trials per experiment. Statistical analyses were conducted using analysis of variance with the SPSS statistical software package (version 18.0; SPSS, Inc., Chicago, IL, USA). Multiple comparisons were performed using the Student–Newman–Keuls method, with statistical significance defined as *P* < 0.05.

## SUPPLEMENTAL MATERIAL

Tables S1 to S3 and Figures S1 to S8 spectrum.01611-24-s0001.docx Supplemental tables and figures.

ASM does not own the copyrights to Supplemental Material that may be linked to, or accessed through, an article. The authors have granted ASM a non-exclusive, world-wide license to publish the Supplemental Material files. Please contact the corresponding author directly for reuse.
