# Supplementary material for: Complete degradation of polycyclic antibiotic methacycline by a micro/nanostructured biogenic Mn oxide composite from engineered Mn(II)-oxidizing Pseudomonas sp. MB04B
Source: Microbiol Spectr. 2025 May 16;13(7):e01611-24. doi: 10.1128/spectrum.01611-24 (PMC12210982; doi:10.1128/spectrum.01611-24)
Supplement: Tables S1 to S3 and Figures S1 to S8 — Supplemental tables and figures. [file spectrum.01611-24-s0001.docx]

**Supporting Material**

**Engineering a micro-/nanostructured biogenic Mn oxide composite by a genetically modified Mn(II)-oxidizing bacterium for complete degradation of methacycline**

Jie Zeng, Zhenghu Tong, Zhi Li, Yongxuan Liu, Li Xie, Tan Wang, Shiwei Li, Lin Li^#^

National Key Laboratory of Agricultural Microbiology, College of Life Science and Technology, Huazhong Agricultural University, Wuhan, China

Running Title: Engineered *Pseudomonas* sp. for degrading methacycline

^#^Address correspondence to Lin Li, lilin@mail.hzau.edu.cn

Jie Zeng and Zhenghu Tong contributed equally to this work. Author order was determined on the basis of seniority.

**Determination of biofilms** The biofilm formation assay was modified from the protocol established by Peeters *et al*. (1). Overnight cultures of the bacterial strains were harvested, and the optical density at 600 nm (OD600) was adjusted to 1.0. The bacterial suspension was then diluted with LB medium at a 1:100 ratio and transferred to a 96-well plate, which was incubated statically at 28°C for 24 hours. Following biofilm formation, the contents were carefully discarded, and the wells were washed twice with water before being fixed at 80°C for 20 minutes. The biofilm was stained with a 1% crystal violet solution at room temperature for 15 minutes, followed by two washes with distilled water. Finally, 200 μL of 95% ethanol was added to each well to solubilize the stain, and the absorbance at 595 nm (OD595) was measured using a microplate reader. Biofilm images were captured using a similar procedure in glass test tubes.

**Determination of c-di-GMP** The pCdrA::*gfp*^C^ fluorescence reporter vector was employed to assess intracellular c-di-GMP levels in the strains. This vector features the fusion of a c-di-GMP response element, the *cdrA* promoter, upstream of the green fluorescent protein (*gfp*) gene. The transcriptional activity of the *cdrA* promoter is modulated by intracellular c-di-GMP concentrations (2, 3). The bacterial strains were cultured overnight, harvested, and the optical density at 600 nm (OD600) was adjusted to 1.0. They were subsequently transferred into LB medium at a 1:100 dilution and incubated at 28°C for 24 h. Intracellular c-di-GMP levels were indirectly quantified by measuring the OD600 and fluorescence intensity at an excitation wavelength of 485 nm and an emission wavelength of 520 nm.

Reference:

1. Peeters E, Nelis HJ, Coenye T. 2008. Comparison of multiple methods for quantification of microbial biofilms grown in microtiter plates. J Microbiol Methods 72:157-165.

2. Borlee BR, Goldman AD, Murakami K, Samudrala R, Wozniak DJ, Parsek MR. 2010. *Pseudomonas aeruginosa* uses a cyclic-di-GMP-regulated adhesin to reinforce the biofilm extracellular matrix. Mol Microbiol 75:827-842.

3. Rybtke MT, Borlee BR, Murakami K, Irie Y, Hentzer M, Nielsen TE, Givskov M, Parsek MR, Tolker-Nielsen T. 2012. Fluorescence-based reporter for gauging cyclic di-GMP levels in *Pseudomonas aeruginosa*. Appl Environ Microbiol 78:5060-5069.

**Supplementary tables**

**Table S1.** Bacterial strains used in this study

| Stains | Characteristics | Sources |
| --- | --- | --- |
| *Escherichia coli* |  |  |
| DH5a | λ-Φ80d*lacZ*ΔM15Δ(*lacZYA*-*argF*)U196 *recA*1 *endA*1, *hsdR*17(rK-, mK -) *supE*44 *thi*-1 *gyrA* *relA*1 | Invitrogen |
| S17-1/λ_pir_ | RP4-2, *pro*-82, λpir*,* *recA*1, *endA*1, *thiE*1, *hsdR*17, *creC*510, donor strain for conjugation | Simon et al., 1983 |
| *Staphylococcus aureus* KCTC 1621 | A wild-type Gram-positive cocci used as a bacterial indicator for bioassay | KCTC ^a^ |
| *Pseudomonas* |  |  |
| MB04B | A wild-type *Pseudomonas* sp. Strain with Mn^2+^-oxidizing activity | Laboratory stock |
| MB04R-14 | A derivative strain of MB04 with joint gene disruption of 7 genes tagged *J6599_19330, J6599_09020, J6599_18500, J6599_10585, J6599_14985, J6599_19340* and *J6599_19360* | This study |
| MB04B*∆00350* | A gene disruptive derivative of MB04B at locus *J6599_00350* | This study |
| MB04B*∆10405* | A gene disruptive derivative of MB04B at locus *J6599_10405* | This study |
| MB04B*∆14985* | A gene disruptive derivative of MB04B at locus *J6599_14985* | This study |
| MB04B*∆09020* | A gene disruptive derivative of MB04B at locus *J6599_09020* | This study |
| MB04B*∆10585* | A gene disruptive derivative of MB04B at locus *J6599_10585* | This study |
| MB04B*∆18500* | A gene disruptive derivative of MB04B at locus *J6599_18500* | This study |
| MB04B*∆19330* | A gene disruptive derivative of MB04B at locus *J6599_19330* | This study |
| MB04B*∆19340* | A gene disruptive derivative of MB04B at locus *J6599_19340* | This study |
| MB04B*∆19360* | A gene disruptive derivative of MB04B at locus *J6599_19360* | This study |
| MB04B*∆06780* | A gene disruptive derivative of MB04B at locus *J6599_06780* | This study |
| *Pseudomonas aeruginosa* ATCC15442 | A wild-type Gram-negative bacterium used as an indicator for bioassay | ATCC ^b^ |

Note: ^a^ KCTC, Korean Collection for Type Cultures; ^b^ ATCC, American Type Culture Collections.

Reference:

Simon, R., Priefer, U., and Pühler, A. (1983) A broad host range mobilization system for in vivo genetic engineering: transposon mutagenesis in Gram negative bacteria. Nat Biotechnol 1: 784−791.

**Table S2**. Plasmids used in this study

| Plasmids | Characteristic | Source or Usage |
| --- | --- | --- |
| pDS3.0 | Suicide plasmid, *bla oriT oriR6K sacB*, Gm^R^ | Gao et al., 2006 |
| pDS3.0_00350 | A derivative of pDS3.0 with insertion of 1964-bp homologous fragment of gene tag *J6599_00350* at *Sac* I site |  |
| pDS3.0_14985 | A derivative of pDS3.0 with insertion of 2292-bp homologous fragment of gene tag *J6599_14985* at *Sac* I site | Disruption of gene tagged *J6599_14985* |
| pDS3.0_10405 | A derivative of pDS3.0 with insertion of 2103-bp homologous fragment of gene tag *J6599_10405* at *Sac* I site | Disruption of gene tagged *J6599_10405* |
| pDS3.0_19330 | A derivative of pDS3.0 with insertion of 2185-bp homologous fragment of gene tag *J6599_19330* at *Sac* I site | Disruption of gene tagged *J6599_19330* |
| pDS3.0_06780 | A derivative of pDS3.0 with insertion of 1972-bp homologous fragment of gene tag *J6599_06780* at *Sac* I site | Disruption of gene tagged *J6599_06780* |
| pDS3.0_10585 | A derivative of pDS3.0 with insertion of 2142-bp homologous fragment of gene tag *J6599_10585* at *Sac* I site | Disruption of gene tagged *J6599_10585* |
| pDS3.0_09020 | A derivative of pDS3.0 with insertion of 1880-bp homologous fragment of gene tag *J6599_09020* at *Sac* I site | Disruption of gene tagged *J6599_09020* |
| pDS3.0_19340 | A derivative of pDS3.0 with insertion of 2120-bp homologous fragment of gene tag *J6599_19340* at *Sac* I site | Disruption of gene tagged *J6599_19340* |
| pDS3.0_18500 | A derivative of pDS3.0 with insertion of 2384-bp homologous fragment of gene tag *J6599_18500* at *Sac* I site | Disruption of gene tagged *J6599_18500* |
| pDS3.0_19360 | A derivative of pDS3.0 with insertion of 2063-bp homologous fragment of gene tag *J6599_19360* at *Sac* I site | Disruption of gene tagged *J6599_19360* |

Reference:

Gao, W., Liu, Y., Giometti, C.S., Tollaksen, S.L., Khare, T., Wu, L. et al. (2006) Knock-out of SO1377 gene, which encodes the member of a conserved hypothetical bacterial protein family COG2268, results in alteration of iron metabolism, increased spontaneous mutation and hydrogen peroxide sensitivity in *Shewanella oneidensis* MR-1. BMC Genomics 7: 76.

**Table S3.** Oligonucleotide primers used in this study

| Primer | Sequence |
| --- | --- |
| AC1 | ACGATGGACTCCAGAG |
| LAD-1 | ACGATGGACTCCAGAGCGGCCGCVNVNNNGGAA |
| LAD-2 | ACGATGGACTCCAGAGCTGCAGGCBNBNNNGGTT |
| LAD-3 | ACGATGGACTCCAGAGCTGCAGGCVVNVNNNCCAA |
| LAD-4 | ACGATGGACTCCAGAGCTGCAGGCBDNBNNNCGGT |
| SP1 | GCTGAGTTGAAGGATCAGATCACGCAT |
| SP2 | ACGATGGACTCCAGTCCGGCCCTCTCATCAACCGTGGCTCCCTCACT |
| SP3 | TTACACTGATGAATGTTCCGTTGCGCTGC |
|  |  |
| J6599_00350-Up | F: 5'−CATGCGATATCGAGCTTGAACTCAGGTTGCAGGGCT−3' |
|  | R: 5'−CATGCGATATCGAGCTTGAACTCAGGTTGCAGGGCT−3' |
| J6599_00350-Down | F: 5'−CATGCGATATCGAGCTTGAACTCAGGTTGCAGGGCT-3' |
|  | R: 5'−CATGCGATATCGAGCTTGAACTCAGGTTGCAGGGCT-3' |
| J6599_14985-Up | F: 5'−CGCATGCGATATCGAGCTGATGCCCGACCCGTTTGT−3' |
|  | R: 5'−CCTATGCGGGGTTCCTTCAGCGAGTGGCTGGCGTTTC−3' |
| J6599_14985-Down | F: 5'−ATGAAACGCCAGCCACTCGCTGAAGGAACCCCGCATAGG−3' |
|  | R: 5'−GGAATTCCCGGGAGAGCTTGCCCCACTTGGTATTGACG−3' |
| J6599_10405-Up | F: 5'−CGCATGCGATATCGAGCTCAGCCGCTGCTTGATGTCC−3' |
|  | R: 5'−CGTCTATCTCCGTGAGCGTTGCGTCGATGACTCCGTTTATTGTTC−3' |
| J6599_10405-Down | F: 5'−GAACAATAAACGGAGTCATCGACGCAACGCTCACGGAGATAGACG−3' |
|  | R: 5'−GGAATTCCCGGGAGAGCTCATGGCGAACGGGAACAG−3' |
| J6599_19330-Up | F: 5'−CGCATGCGATATCGAGCTTGCGAAGAGGATGCCCAGTG−3' |
|  | R: 5'−ATTGTCCGCCTCACGCCCAAGTACCAGGGTGCGGTGCTG−3' |
| J6599_19330-Down | F: 5'−CAGCACCGCACCCTGGTACTTGGGCGTGAGGCGGACAAT−3' |
|  | R: 5'−GGAATTCCCGGGAGAGCTTGCAGAGCCGGGTAGAGCAG−3' |
| J6599_06780-Up | F: 5'−CGCATGCGATATCGAGCTGCAGCGTGGAGCCGGTAAT-3' |
|  | R: 5'−AATTTGCCATCATTGCCGCGGCGGTGCTTGGGAGGAAAT−3' |
| J6599_06780-Down | F: 5'−ATTTCCTCCCAAGCACCGCCGCGGCAATGATGGCAAATT−3' |
|  | R: 5'−GGAATTCCCGGGAGAGCTCCGTCCCAGCAGGTAACCCT−3' |
| J6599_10585-Up | F: 5'−CGCATGCGATATCGAGCTAGCACCACCAACCTGTTCCG-3' |
|  | R: 5'−TTTACTCCGCTTTGGCGTCTGCTGCCACAGCCCTTGATTAGC−3' |
| J6599_10585-Down | F: 5'−GCTAATCAAGGGCTGTGGCAGCAGACGCCAAAGCGGAGTAAA−3' |
|  | R: 5'−GGAATTCCCGGGAGAGCTCGGAGTGGTGTCATACGGGAAT−3' |
| J6599_09020-Up | F: 5'−CGCATGCGATATCGAGCTTCCACGGTCGCACCCTGTT−3' |
|  | R: 5'−AAACTCTGCTAACGCATCAATCCCGACGGCGGCACGGATAAA−3' |
| J6599_09020-Down | F: 5'−TTTATCCGTGCCGCCGTCGGGATTGATGCGTTAGCAGAGTTT−3' |
|  | R: 5'−GGAATTCCCGGGAGAGCTTGCGGTTGTGGATGATGAAAGG−3' |
| J6599_19340-Up | F: 5'−CGCATGCGATATCGAGCTGGTGGTCCTTGTTCTGAATCTGG−3' |
|  | R: 5'−ACTCCCTCCCTATACTTGTCTCCACCTTACTGTTTACCGTTTGCCTGA−3' |
| J6599_19340-Down | F: 5'−TCAGGCAAACGGTAAACAGTAAGGTGGAGACAAGTATAGGGAGGGAGT−3' |
|  | R: 5'−GGAATTCCCGGGAGAGCTACCTGAGCCATGCGATCAAC−3' |
| J6599_18500-Up | F: 5'−CGCATGCGATATCGAGCTTGCTCGGTCAGGCTCAGTTT−3' |
|  | R: 5'−GGGTGCTGCGTTTCACGTTCAGTGCGGTATTCACGGTCAGTTC−3' |
| J6599_18500-Down | F: 5'−GAACTGACCGTGAATACCGCACTGAACGTGAAACGCAGCACCC−3' |
|  | R: 5'−GGAATTCCCGGGAGAGCTGCAAATCGGAACGACGGCATA−3' |
| J6599_19360-Up | F: 5'−CGCATGCGATATCGAGCTTTCGACACGCAACAGCACG−3' |
|  | R: 5'−GAAGTTCGTAATGTCAGCAAGCCGAAGGTTTGGCAGAAGGTG−3' |
| J6599_19360-Down | F: 5'−CACCTTCTGCCAAACCTTCGGCTTGCTGACATTACGAACTTC-3' |
|  | R: 5'−GGAATTCCCGGGAGAGCTCTGATGCTGGTGGTTTCCTT−3' |

**Supplementary figures**


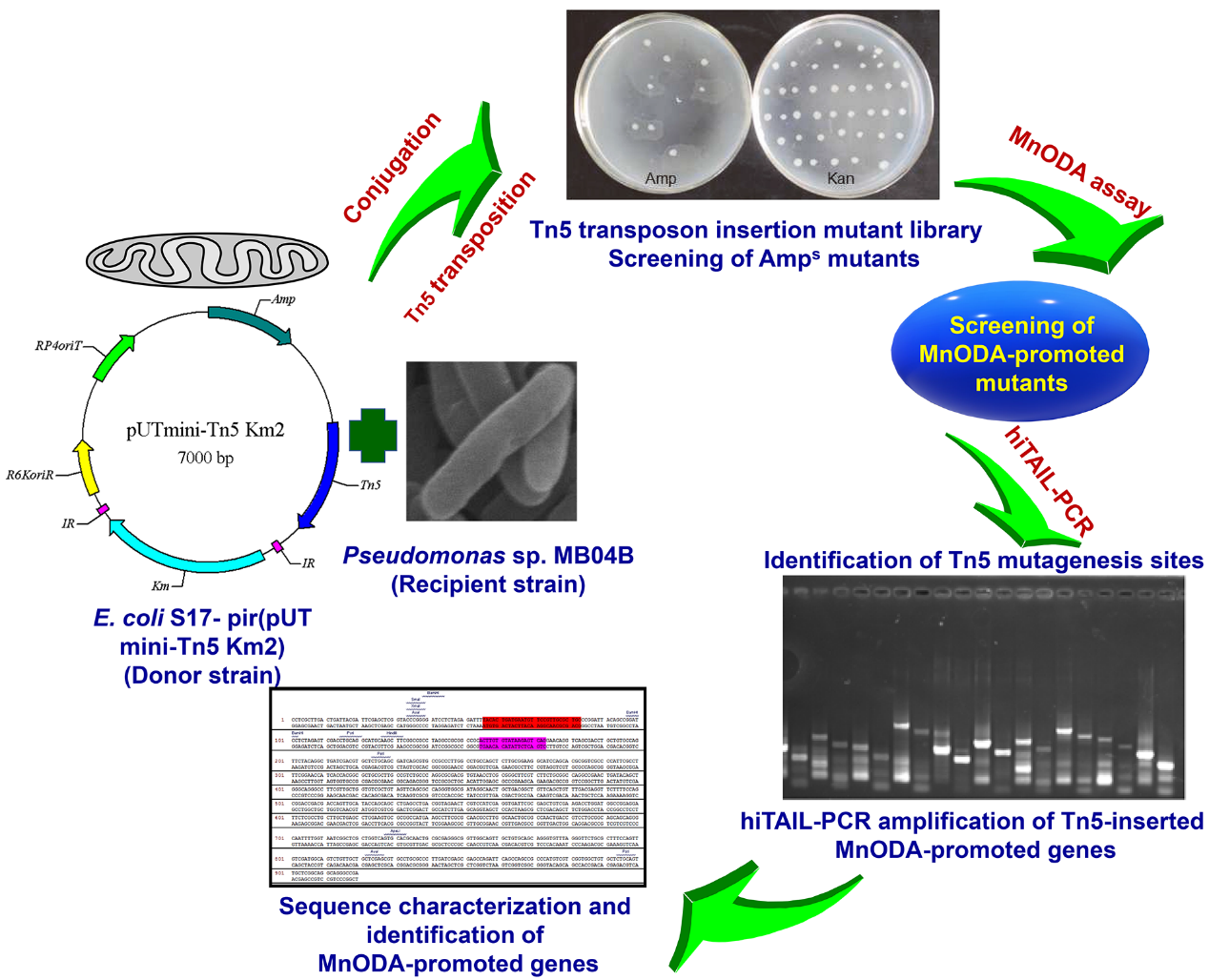


**Fig. S1.** Schematic illustration of the construction of mini-Tn5 transposon mutant library and identification of mini-Tn5 mutagenesis sites and target genes involving in the promotion of Mn(II)-oxidizing activity in *Pseudomonas* sp. MB04B.

**Fig. S2.** Measurement of MnODA in 10 mini-Tn5 mutagenesis. Means followed by different letters in a column are significantly different (*p* < 0.05) according to the SNK test.


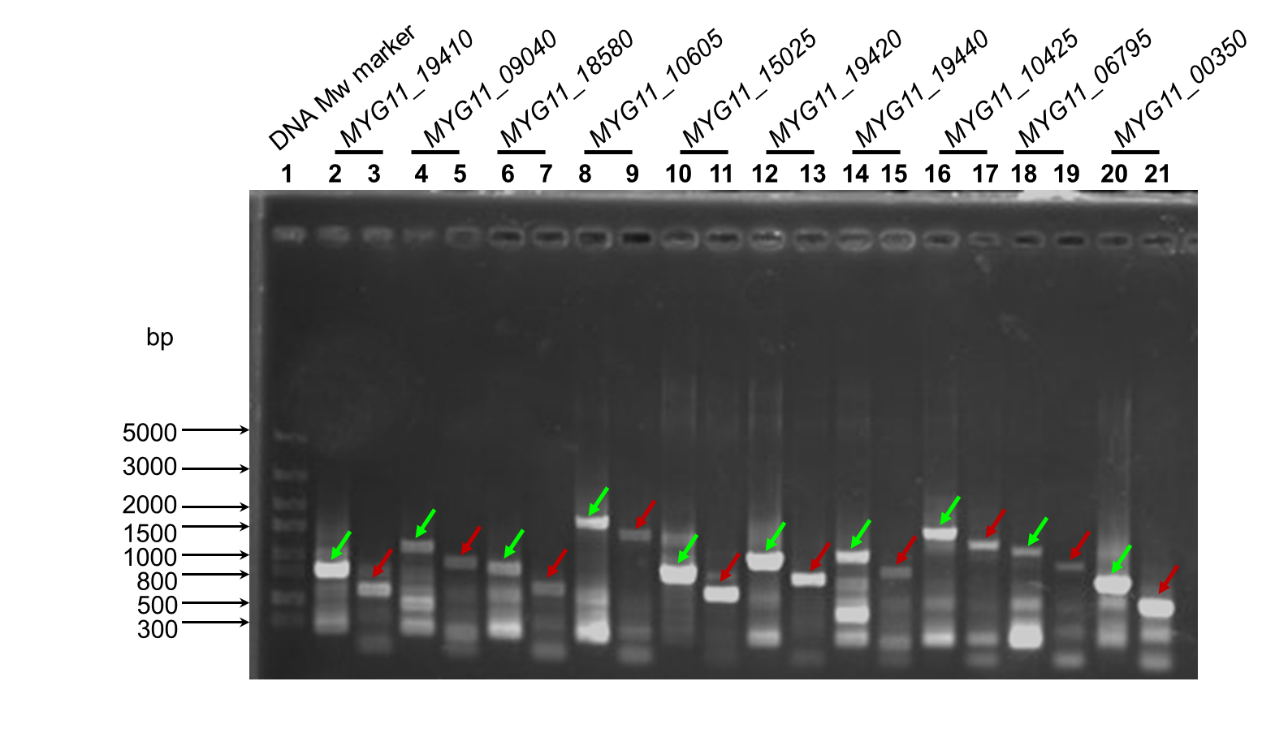


**Fig. S3.** Electrophoresis profile of the amplified products by hiTAIL-PCR. Lane 1, DNA marker trans5K; lanes 2/4/6/8/10/12/14/16/18/20 and lanes 3/5/7/9/11/13/15/17/19/21, the second-round (As shown by green arrow) and the third-round (As shown by red arrow) amplified products of flanking sequences of mini-Tn5 inserted gene loci of *MYG11_19330*, *MYG11_09020*, *MYG11_18500*, *MYG11_10585*, *MYG11_14985*, *MYG11_19340*, *MYG11_19360*, *MYG11_10405*, *MYG11_06780* and *MYG11_00350*, respectively.


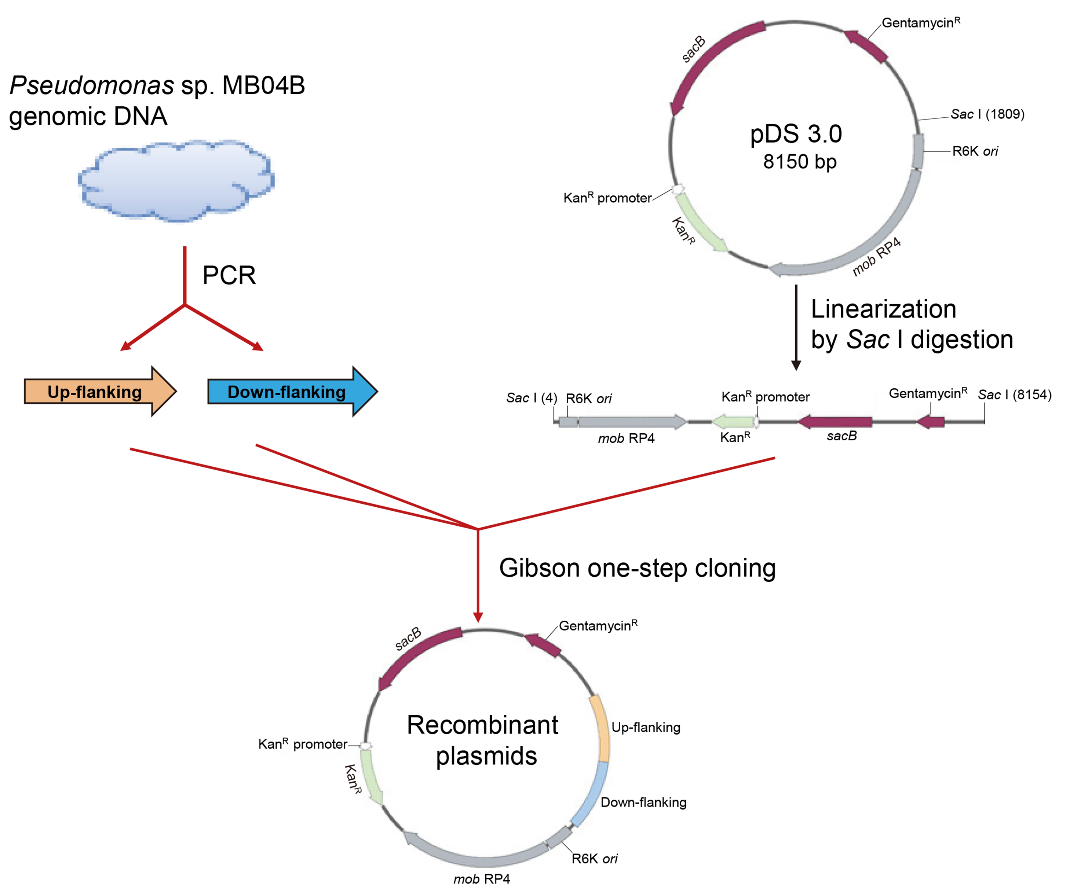


**Fig. S4.** Technical illustration of constructing recombinant plasmids for the knockout of target genes in *Pseudomonas* sp. MB04B chromosome.


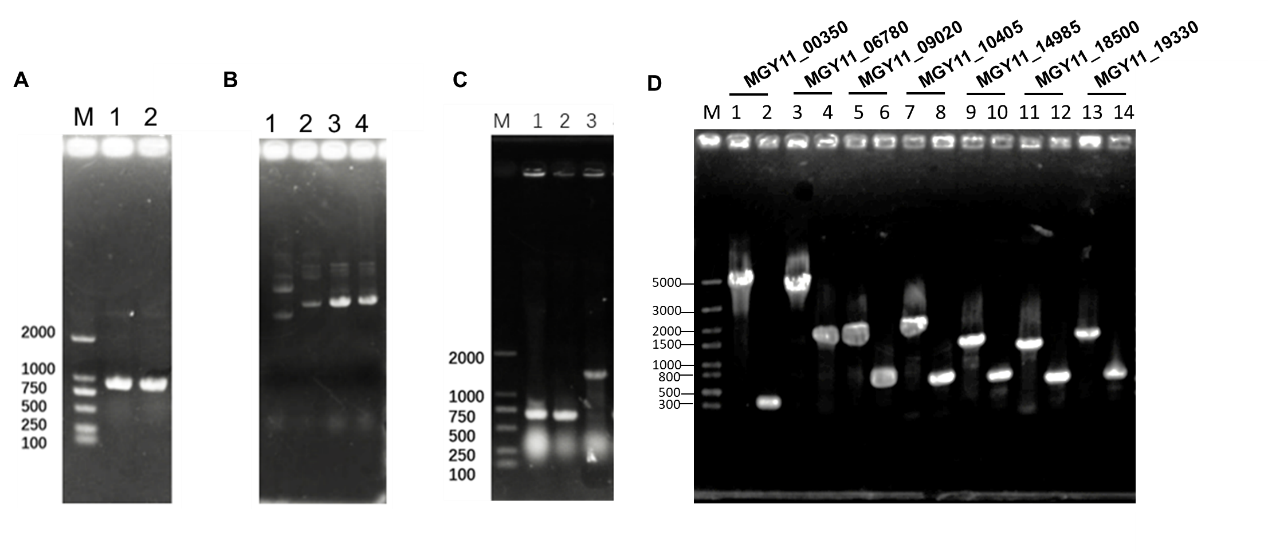


**Fig. S5**. Identification of gene-disruptive mutant strains through cross-checked PCR-amplified products using different primer combinations. A, PCR amplified products of homologous arms; B, from Gene-disruptive vectors. Lane 1, from parent vector plasmid, lane 2/3/4, from successful disruptive strains; C, verification of the gene *MGY11_09020* knockout. Lane 1/2, successful knockout, lane 3, negative control. D, Verification of mutant strains with multiple gene disruptions. Lanes 1/3/5/7/9/11/13 and lanes 2/4/6/8/10/12/14 indicate parent and disruptive fragments of the genes *MGY11_00350*, *MGY11_06780*, *MGY11_09020*, *MGY11_10405*, *MGY11_14985*, *MGY11_18500* and *MGY11_19330*, respectively.

**Fig. S6.** Measurement of MnODA for the intermediate mutants (from the single knockout to the seven-gene knockout strains). The knockout sequence is as follows: *MYG11_00350, MYG11_15025, MYG11_09040, MYG11_19410, MYG11_18580, MYG11_10425, MYG11_06795.* Means followed by different letters in a column are significantly different (*p* < 0.05) according to the SNK. test.


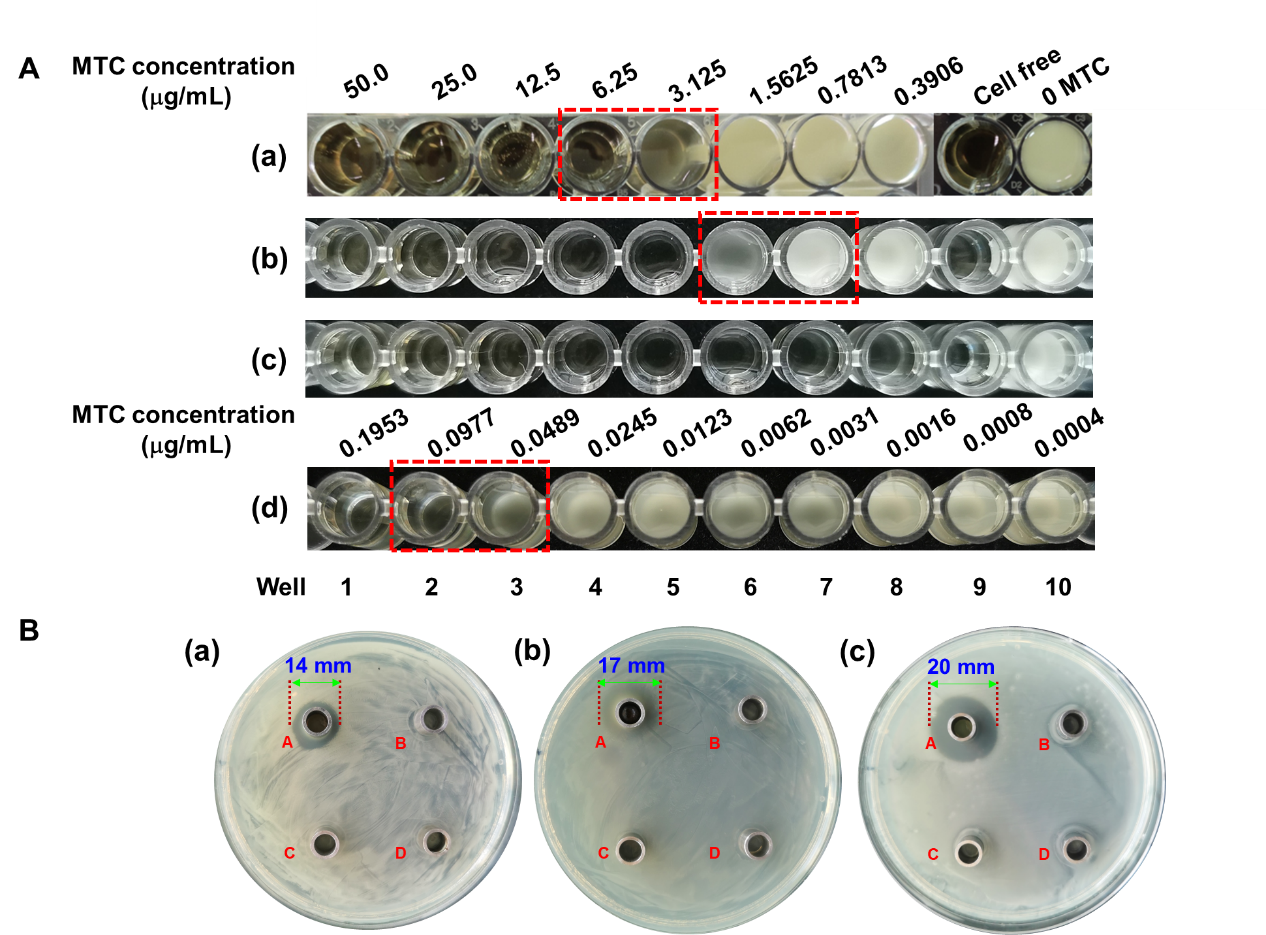


**Fig. S7**. A. MIC assay of *E. coli* DH5α (a), *P. aeruginosa* ATCC15442 (b), and *S. aureus* KCTC1621 (c and d). B. Antibacterial zone test of the MTC-degraded products by the BMO composite against *E. coli* DH5α (a), *P. aeruginosa* ATCC15442 (b), and *S. aureus* KCTC1621 (c). In A(a) to A(c), well 1 to well 8, loaded 2-times diluted MTC at the initial MTC concentration of 50 μg/mL; well 9, 50 μg /mL MTC and cell free; well 10, 0 MTC with test strain. In A(d), well 1 to well 10, loaded 2-times diluted MTC at the initial MTC concentration of 0.1953 g/mL. In B, cup A, with 50 μg /mL MTC; cup B, with MTC-degraded product by the BMO composite; cup C, with 2-times diluted MTC-degraded product by the BMO composite; cup D, with three-times diluted MTC-degraded product by the BMO composite.


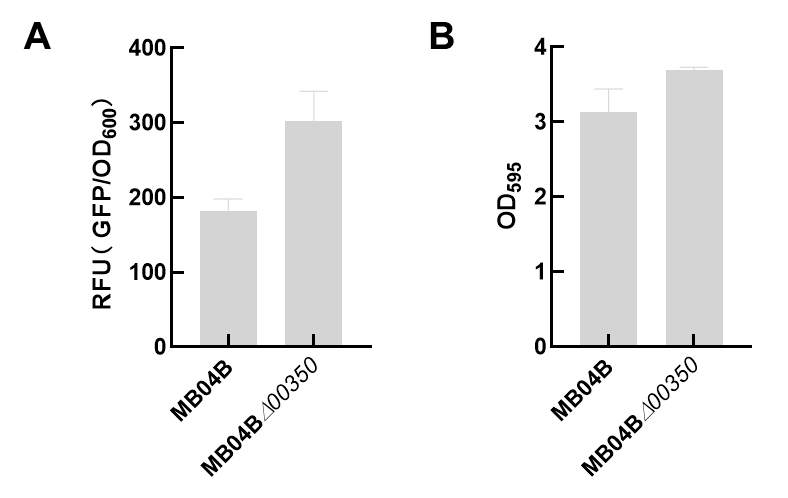


**Fig. S8.** Biofilm formation (A) and c-di-GMP level (B) of MB04B and MB04B△*00350*
